# Interleaved quartic high gain DC–DC converter

**DOI:** 10.1038/s41598-024-84015-w

**Published:** 2025-01-02

**Authors:** T. Sakthiram, L. Yogesh, Rahul Srikanth, M. Prabhakar, Kazem Varesi

**Affiliations:** 1 School of Electrical Engineering, Vellore Institute of Technology, Chennai Campus, Vellore, India; 2 Centre for Smart Grid Technologies, Vellore Institute of Technology, Chennai Campus, Vellore, India; 3 Power Electronics Research Laboratory (PERL), Faculty of Electrical Engineering, Sahand University of Technology, Tabriz, Iran

**Keywords:** DC–DC converters, Power conversion, Power electronics, Microgrids, Energy science and technology, Engineering

## Abstract

This research paper presents a high-gain DC–DC converter with ultra-step-up voltage gain capability. The proposed converter is synthesized from a two-phase interleaved boost converter (IBC), and its voltage gain is doubled by adopting a voltage lift capacitor. To enhance its voltage gain capability, a floating capacitor-based gain extension cell is adopted subsequently. This cell yields a voltage gain that is cubed times the output voltage obtained from a classical boost converter (CBC). By cascading the two stages, the voltage gain of the proposed converter is enhanced to quartic times (4th power) that of the CBC. The proposed gain extension concept is validated by conducting practical experiments on a 16 V to 400 V, 150 W prototype version. Practically, the prototype converter delivers 150 W to the load and operates at a full-load efficiency of 92.7% when its switches are operated at safe duty ratio values. Under dynamic conditions, the proposed converter regulates the output voltage to 400 V quickly over a wide range of input voltage and load current variations; the overshoots and undershoots are also negligible. The maximum voltage gain of the proposed converter momentarily increases to 37 when the input voltage is drastically reduced to 10.8 V while the switches are still operated at safe duty ratio values. The voltage stress on the semiconductor devices is only a fraction of the output voltage due to the hybrid voltage gain extension technique. The input current is also ripple-free as the switches in the IBC structure are always operated at a duty ratio of 50%, and only the third switch is controlled to meet the required voltage gain. The salient features of the proposed converter are clearly highlighted by comparing it with several converters that possess quadratic, cubic, and quartic voltage gain functions. The common-ground connection between the source and the load in the proposed converter is an added preferable feature for PV applications.

## Introduction

The escalating energy demands, driven by the proliferation of industrial loads, electric vehicles, data centres, etc., underscore the urgency of transitioning to renewable energy sources (RES) for curbing carbon emissions. Solar energy emerges as a prime candidate, given its widespread availability and perpetual abundance^1^. However, photovoltaic (PV) generation typically operates at low voltage levels (12–60 V), posing challenges when integrating with the standard 380 V DC grid. This integration often necessitates high-gain power electronic converters^2^.

High-gain converters (HGCs) are crucial for achieving elevated voltage levels. Classical boost converters (CBCs) face limitations in high-gain applications, prompting the adoption of gain extension techniques like voltage multiplier cells (VMCs). While VMCs offer higher voltage gain and reduced voltage stress on the switch, their effectiveness is constrained by additive voltage amplification, necessitating additional components^3–7^. Furthermore, impulsive charging susceptibility in power switches and diodes compromises the efficiency of VMC-based solutions. These challenges highlight the imperative need to adopt innovative approaches for efficiently integrating the low-voltage PV panels into the high-voltage grids.

Coupled inductors (CIs) are employed within the CBC structure to achieve a high voltage gain ratio, which can be easily extended by adjusting the turns ratio^8–10^. In^8–10^, the voltage lift technique is adopted along with complementary switching to achieve twice the voltage gain of CBC with ripple-free input current. Employing a diode capacitor multiplier (DCM) network integrated with CIs and VMCs achieves significant voltage amplification in^11^. However, its scalability is still limited due to the linear gain obtained.

To achieve high voltage amplification while minimizing voltage stress, power circuit topologies combine switched capacitors (SCs) and switched inductors (SIs) with CBC^12,13^. The transition between series and parallel operation in SIs inherently causes pulsating input currents. To further enhance the efficiency of switched inductor networks, an inductor-capacitor-inductor (LCL) network is employed, extending voltage gain at the expense of LC oscillations^14–16^. Although the converter in^14^ achieves an impressive voltage gain amplification of 12.7, it operates at a high duty ratio of 0.67 due to the linear voltage gain profile. Another notable drawback is that most of the power topologies employing SIs often utilize one power switch that experiences high current stress more than the input current. Hence, there is a need for topologies with higher-order voltage amplification.

The converter in^17^ utilizes a unique combination of SCs and SIs with dual switches to achieve a higher-than-quadratic voltage gain level at reduced switch stress magnitudes. However, limitations in voltage regulation, relying solely on the number of SC cells, are its main drawbacks.

Quadratic boost converters (QBCs) are combined with existing gain extension techniques such as SCs and SIs to further extend the voltage gain of the SI-based converters^18–20^. By adopting active SI-based topologies, component count is reduced. However, a notable drawback lies in the increased complexity of driving power MOSFETs with a floating ground. In^21^, a hybrid quadratic converter is presented. Despite offering a high voltage gain value, the voltage stress on the diodes used in the gain extension cells is higher.

In^22^, a converter with a quadratic voltage gain function is described. The converter shares a common ground between the input and the output. The converter described in^23^ adopts a hybrid QBC with an embedded SC and VMC to achieve enhanced quadratic voltage amplification. In^24^, a modified QBC featuring floating power switches and an energy recycling scheme using SCs is introduced. The innovative scheme enables remarkable voltage amplification while simultaneously reducing the current stress on inductors. Similar approaches that embed QBCs with existing gain extension mechanisms like VMCs or SCs achieve substantial voltage amplification while minimizing the voltage stress are detailed in^25,26^.

Generally, CI-based converters, when paired with suitable gain extension techniques, meet the high-voltage gain needs effectively. When CIs are implemented in QBC-based structures, impressive voltage amplification profiles are obtained^27–34^. In such structures, hybrid combinations of gain extension techniques are adopted, viz., CI-SC^28^, CI with DCMs^29^, QBC with SC^30^, and CI-VMC^31^, to mainly reduce the voltage stress on the switches. In^32^, a QBC equipped with a three-winding CI is introduced. An impressive voltage amplification of 20 at a secured duty ratio of 0.56 is obtained while requiring smaller-sized inductors only. However, addressing factors like size, weight, and leakage inductance is crucial for optimal performance. Additionally, implementing clamping circuitry to mitigate voltage spikes caused by leakage inductance adds to their design complexity. Thus, single-switch higher-order converters, like cubic or quartic boost converters, present alternative solutions.

In^33^, a modular single-switch cubic boost converter (SS-C^3^BC) with modified diode-inductor-capacitor (DLC) voltage-boosting cells is proposed. The converter in^34^ adopts the SS-C^3^BC structure and is cascaded with a VMC to achieve ultra-high voltage gain. The voltage stress on the switch is also reduced due to the adopted structure. The converters in^35,36^ employ an energy recycling scheme to reduce the voltage rating of the capacitors besides achieving cubic voltage gain capability.

Intriguingly, the converter presented in^37^ introduces a novel single-switch bi-quadratic boost design and employs a switched inductor-capacitor network (SLCN) to attain an excellent voltage gain characteristic; the voltage gain is quartic times (fourth power of) the CBC’s voltage gain. Nevertheless, the switch is subjected to an increased voltage stress due to its proximity to the output. Additionally, the input current ripple is also on the higher side. In all the single-switch QBCs presented in^33–37^, the power handling capability is constrained as the switch bears a higher current load than the input current. To enhance the power handling capability, higher-order converters often employ multiple-parallel-operated power switches.

Evidently, IBCs operate two CBCs in parallel to reduce the input current stress and ripple. Interleaving multiple QBC structures effectively minimizes the input current ripple. In^38^, two QBC structures are interleaved to create an interleaved QBC (IQBC); coupling its two phases with a voltage-lift capacitor yields a remarkable voltage gain of 16.66. Nevertheless, the voltage gain falls short of single-switch converters that employ the same number of components. In^39^, cubic voltage gain functionality is achieved by using multiple power switches. Cubic boost converters (C^3^BC) are created by integrating IBC with QBC structures, enabling cubic voltage gain functionality. Despite using many components, the converter operates at high efficiency values even at elevated voltage conversion ratios, besides drawing smooth current from the input.

In this manuscript, a high gain converter with a quartic voltage conversion ratio profile is presented. The main contributions of this paper are (i) introducing a novel gain extension cell, (ii) synthesizing a floating-capacitor-based converter stage with cubic voltage gain capability, and (iii) synthesizing a converter that possesses quartic voltage gain functionality. This paper is articulated as follows: In “Introduction” section, existing converters are thoroughly reviewed and the proposed converter is introduced. “Power circuit and its operating principle” section describes the power circuit configuration and its operating principle. The design expressions are derived from basic principles and presented in “Voltage conversion and design equations” section. In “Experimental results and discussion” section, the experimental results obtained from a laboratory prototype converter are described along with the inferences. “Benchmarking the proposed converter” section presents a detailed comparison between the proposed converter and other similar converters that are available in the literature. Finally, the concluding remarks are presented, and the manuscript is wrapped up.

## Power circuit and its operating principle

### Description of the power circuit

**Fig. 1:**
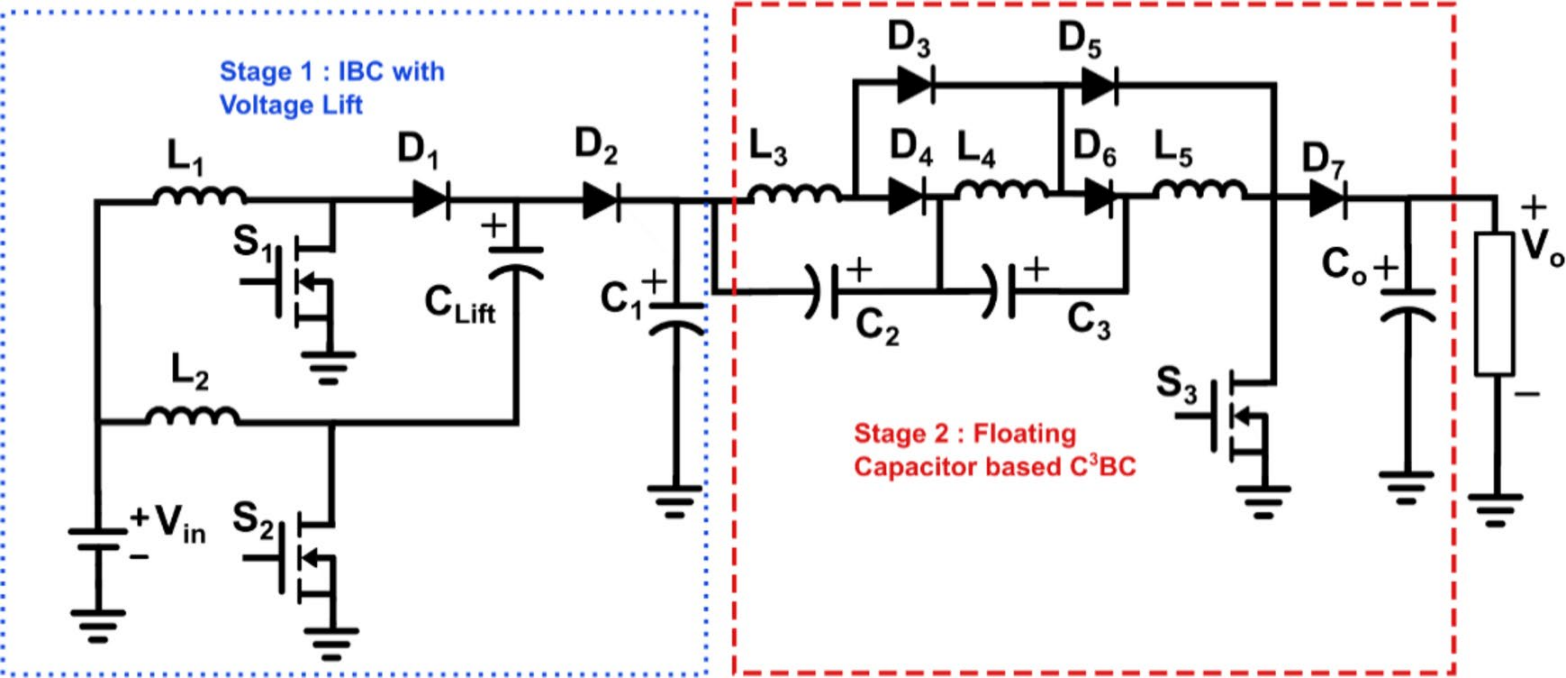
Power circuit diagram of the proposed quartic high gain converter (Q^4^HGC).

Figure 1 portrays the power circuit of the proposed quartic high-gain converter (Q^4^HGC). The proposed converter is synthesized by cascading two different stages. Stage 1 comprises a two-phase IBC coupled with a voltage lift capacitor *C*_*Lift*_. The output from stage 1 is coupled to stage 2 through the capacitor *C*_*1*_. Stage 2 is synthesized using discrete inductors *L*_*3*_, *L*_*4*_, and *L*_*5*_, which act as the classical energy storage inductors in boost-derived topologies. They aid in enhancing the voltage gain obtained from their respective previous stages. The negative plates of capacitors *C*_*2*_ and *C*_*3*_ are connected to the positive plates of *C*_*1*_ and *C*_*2*_ respectively. The series combination of capacitors aids in charging the inductors and obtaining higher voltage levels. Thus, the voltage gain developed in stage 2 is split across the intermittent capacitors *C*_*2*_ and *C*_*3*_. The discrete inductors, capacitors, and the diodes *D*_*3*_-*D*_*6*_, along with the single switch *S*_*3*_ are carefully synthesized to operate as a floating capacitor cubic cell (F-C^3^BC). Diode *D*_*7*_ acts as a boost rectifier diode in a CBC, and *C*_*0*_ is the output filter capacitor.

### Operating principle

In the proposed Q^4^HGC, *S*_*1*_ and *S*_*2*_, located in stage 1, are operated with a fixed duty ratio of *δ*_*1*_ = *δ*_*2*_ = 0.5 and with a phase-shift of 180° to obtain a ripple-free input current. The operation of stage 2 is controlled by *S*_*3*_, and its operation is independent of *S*_*1*_ and *S*_*2*_. Nevertheless, the operation of *S*_*3*_ is synchronized with *S*_*1*_ for easier control of the proposed Q^4^HGC. The following valid assumptions are made to easily understand the operating principle.

(i) All the switching elements are ideal.
(ii) The duty ratio of *S*_*3*_ is less than that of *S*_*1*_ (and *S*_*2*_), i.e., δ_3_ < δ_2_.
(iii) The converter draws continuous current from the input, and all the inductors operate in continuous conduction mode (CCM).
(iv) All the inductors are precharged.

#### Mode 1 (0 < t < t1)

This mode commences when *S*_*1*_ and *S*_*3*_ are turned ON at t = *t*_*0*_. The discrete inductor *L*_*1*_ charges linearly through the *S*_*1*_. Since the switch *S*_*2*_ is OFF, inductor *L*_*2*_ discharges and transfers its stored energy to capacitor *C*_*1*_ through *C*_*Lift*_ and *D*_*2*_. Diode *D*_*1*_ is reverse-biased as *S*_*1*_ is conducting. In stage 2, the currents through the inductors *L*_*3*_, *L*_*4*,_ and *L*_*5*_ linearly rise through the diodes *D*_*3*_, *D*_*5*,_ and *S*_*3*_ respectively. The energy stored in *C*_*1*_ is transferred to *L*_*3*_, *D*_*3*,_*D*_*5*,_ and *S*_*3.*_ The energy stored in *L*_*4*_ begins to rise linearly towards the potential across the series combination of *C*_*1*_ and *C*_2_.

**Fig. 2:**
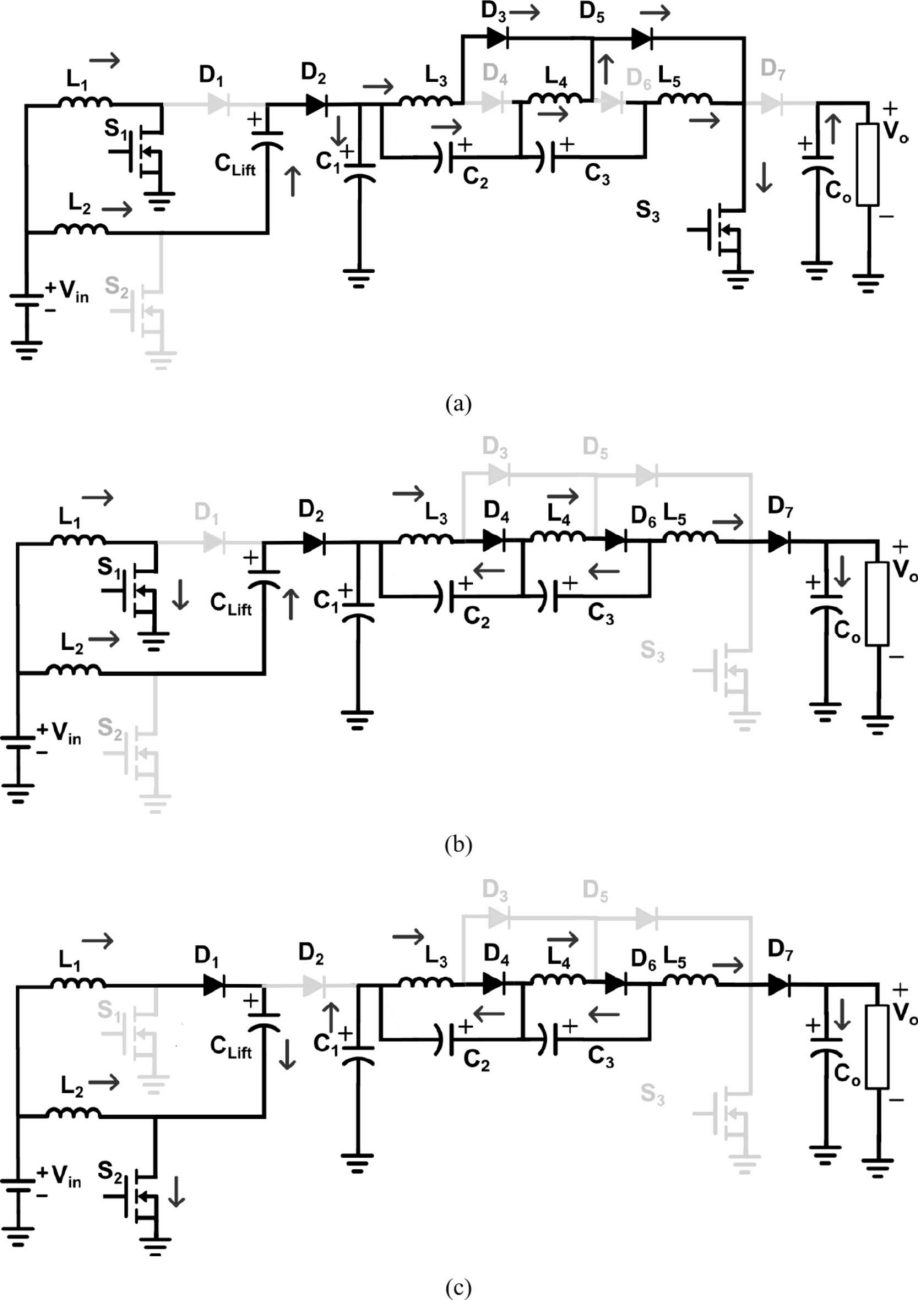
Equivalent circuit of proposed Q^4^HGC in (**a**) Mode 1, (**b**) Mode 2, and (**c**) Mode 3.

Similarly, *L*_*5*_ is also charged by the effective series combination of *C*_*1*_, *C*_*2*,_ and *C*_*3*_. Diodes *D*_*4*_ and *D*_*6*_ are reverse-biased as the discrete inductors remain in charging condition. Since *D*_*7*_ acts like the boost-rectifier diode, it also remains in the reverse-biased state as *S*_*3*_ is conducting and the output capacitor *C*_*0*_ supplies the load requirement. Mode 1 ends at t = *t*_*1*_ when the current through *L*_*3*_, *L*_*4*_ and *L*_*5*_, reaches their respective maximum values${I_{{L_3},\hbox{max} ,}}{\text{ }}{I_{{L_4},\hbox{max} ,}}{\text{ and }}{I_{{L_5},\hbox{max} ,}}$. The equations governing this mode of operation are given by (1)–(7), while the equivalent circuit during Mode 1 is depicted in Fig. 2(a).

1 $${i_{{L_1}}}\,={I_{{L_1},\hbox{min} }}\,+\,\,\,\frac{{{V_{{L_1}}}}}{{{L_1}}}t\,\,\,\,\,={I_{{L_1},\hbox{min} }}\,+\,\,\,\frac{{{V_{in}}}}{{{L_1}}}t$$

2 $${i_{{L_2}}}\,={I_{{L_2},\hbox{max} }}\,+\,\,\,\frac{{{V_{{L_2}}}}}{{{L_2}}}t\,\,\,\,\,={I_{{L_2},\hbox{max} }}\, - \,\,\,\frac{{({V_{in}}+{v_{{C_{Lift}}}} - {v_{{C_1}}})}}{{{L_2}}}t$$

3 $${i_{{L_3}}}\,={I_{{L_3},\hbox{min} }}\,+\,\,\,\frac{{{V_{{L_3}}}}}{{{L_3}}}t\,\,\,\,\,={I_{{L_3},\hbox{min} }}\,+\,\,\,\frac{{{v_{{C_1}}}}}{{{L_3}}}t$$

4 $${i_{{L_4}}}\,={I_{{L_4},\hbox{min} }}\,+\,\,\,\frac{{{V_{{L_4}}}}}{{{L_4}}}t\,\,\,\,\,={I_{{L_4},\hbox{min} }}\,+\,\,\,\frac{{{v_{{C_1}}}+{v_{{C_2}}}}}{{{L_4}}}t$$

5 $${i_{{L_5}}}\,={I_{{L_5},\hbox{min} }}\,+\,\,\,\frac{{{V_{{L_5}}}}}{{{L_5}}}t\,\,\,\,\,={I_{{L_5},\hbox{min} }}\,+\,\,\,\frac{{{v_{{C_1}}}+{v_{{C_2}}}+{v_{{C_3}}}}}{{{L_5}}}t$$

6 $${v_{{C_x}}}=\,{V_{{C_x},\hbox{min} \,}} - \,\frac{{{i_{{C_x}}}}}{{{C_x}}}t\,\,where\,x=1,2,3,4$$

7 $${v_{{C_{Lift}}}}=\,{V_{{C_{Lift}},\hbox{min} \,}}+\,\,\frac{{{i_{{C_{Lift}}}}}}{{{C_{Lift}}}}t\,\,$$

#### Mode 2 (t1 < t < t2)

Mode 2 commences at t = *t*_*1*_ when *S*_*3*_ is turned OFF while *S*_*1*_ continues to remain in the ON state. Inductor *L*_*1*_ continues to charge through *S*_*1*_ while *L*_*2*_ continues to charge *C*_*1*_. Since *S*_*3*_ is turned OFF, due to the electrical inertia of *L*_*5*_, *D*_*7*_ is forward-biased. Likewise, *D*_*4*_ and *D*_*6*_ are also forward-biased due to the electrical inertia of the inductors *L*_*3*_ and *L*_*4*_, respectively.

Simultaneously, *D*_*3*_ and *D*_*5*_ are reverse-biased. The discrete *L*_*3*_ and *L*_*4*_ transfer their stored energy into capacitors *C*_*2*_ and *C*_*3*_ through the *D*_*4*_ and *D*_*6*_, forming independent loops. The inductor *L*_*5*_ transfers its stored energy and charges capacitor *C*_*0*_ through *D*_*7*_ besides meeting the load requirement. Mode 2 comes to an end at t = *t*_*2*_ when the currents through *L*_*1*_ and *L*_*2*_ attain their respective maximum and minimum values given by ${I_{{L_1},\hbox{max} ,}}{\text{ and }}{I_{{L_2},\hbox{min} }}$. The equations governing stage 1 remain unchanged, while the equations governing stage 2 are given by (8)–(11). The equivalent circuit during Mode 2 is portrayed in Fig. 2(b).

8 $${i_{{L_3}}}\,={I_{{L_3},\hbox{max} }}\, - \,\,\,\frac{{{V_{{L_3}}}}}{{{L_3}}}t\,\,\,\,\,={I_{{L_3},\hbox{max} }}\, - \,\,\,\frac{{{v_{{C_2}}}}}{{{L_3}}}t$$

9 $${i_{{L_4}}}\,={I_{{L_4},\hbox{max} }}\, - \,\,\,\frac{{{V_{{L_4}}}}}{{{L_4}}}t\,\,\,\,\,={I_{{L_4},\hbox{max} }}\, - \,\,\,\frac{{{v_{{C_3}}}}}{{{L_4}}}t$$

10 $${i_{{L_5}}}\,={I_{{L_5},\hbox{max} }}\, - \,\,\,\frac{{{V_{{L_5}}}}}{{{L_5}}}t\,\,\,\,\,={I_{{L_5},\hbox{max} }}\, - \,\,\,\frac{{({V_0} - {v_{{C_1}}} - {v_{{C_2}}} - {v_{{C_3}}})}}{{{L_4}}}t$$

11 $${v_{{C_x}}}=\,{V_{{C_x},\hbox{min} \,}}+\,\frac{{{i_{{C_x}}}}}{{{C_x}}}t\,\,where\,x=2,3,4$$

#### Mode 3 (*t*_*2*_ < t < T)

**Fig. 3:**
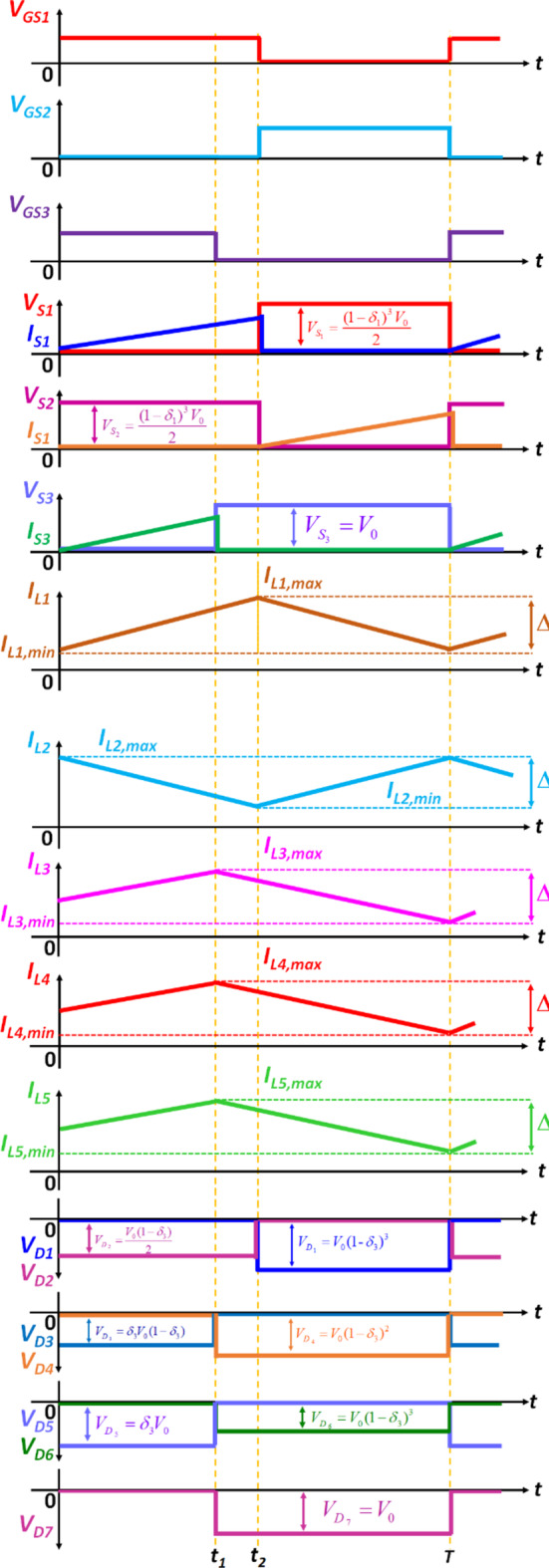
Characteristic waveforms of the proposed Q^4^HGC.

Mode 3 operation begins when *S*_*1*_ is turned ON while *S*_*2*_ and *S*_*3*_ continue to be turned OFF. Due to electrical inertia, *L*_*1*_ forward-biases *D*_*1*_ and transfers its energy to *C*_*Lift*_ while *L*_*2*_ linearly charges through *S*_*2*_. In stage 2, diodes *D*_*4*_, *D*_*6*,_ and *D*_*7*_ remain forward-biased due to the energy stored in the inductors *L*_*3*_, *L*_*4*,_ and *L*_*5*_. Eventually, the inductors *L*_*3*_, *L*_*4*,_ and *L*_*5*_ reach their minimum energy levels (${I_{{L_3},\hbox{min} ,}}{\text{ }}{I_{{L_4},\hbox{min} ,}}{\text{ and }}{I_{{L_5},\hbox{min} }}$) while the capacitors *C*_*2*_, *C*_*3*,_ and *C*_*0*_ reach their maximum energy level. Simultaneously, current through *L*_*2*_ reaches its maximum value ${I_{{L_2},\hbox{max} }}$. Thus, at time t = *T*, one switching cycle is completed when *S*_*1*_ and *S*_*3*_ are turned ON again while *S*_*2*_ is turned OFF. In Mode 3, the equations governing stage 2 are the same as in (8)–(11). The equations for stage 1 during Mode 3 are given by (12), (13). The equivalent circuit during Mode 3 is shown in Fig. 2(c). The characteristic waveforms of the proposed Q^4^HGC are depicted in Fig. 3.

12 $${i_{{L_1}}}\,={I_{{L_1},\hbox{max} }}\, - \,\,\frac{{{V_{{L_1}}}}}{{{L_1}}}t\,\,\,\,\,={I_{{L_1},\hbox{min} }}\, - \,\,\,\frac{{({v_{{C_{Lift}}}} - {V_{in}})}}{{{L_1}}}t$$

13 $${i_{{L_2}}}\,={I_{{L_2},\hbox{min} }}\,+\,\,\,\frac{{{V_{{L_2}}}}}{{{L_2}}}t\,\,\,\,\,={I_{{L_2},\hbox{min} }}\,+\,\,\,\frac{{{V_{in}}}}{{{L_2}}}t$$

## Voltage conversion and design equations

In this section, the voltage conversion ratio of the proposed Q^4^HGC, the voltage and current stress for the switching elements, and the passive elements are derived.

### Voltage conversion ratio

The voltage gain expression of the proposed quartic boost converter is derived by applying voltage-second balance across the inductors. The voltage gain expression is also intuitively understood by considering gain across different stages in the proposed converter.

The first stage consists of IBC with voltage-lift technique that provides twice the voltage gain of a CBC across the capacitor *C*_*1*_ and is given by (14).

14 $${M_{Stage1}}=\,\frac{{{V_{{C_1}}}}}{{{V_{in}}}}\,=\,\frac{2}{{(1 - {\delta _1})}}$$

The second stage consists of a floating capacitor-based cubic cell boost converter. The capacitor *C*_*1*_ acts like a stiff source for this stage. Thus, the cumulative voltage gain is obtained as a product of the voltage amplification obtained across stage 1 and stage 2. However, since the capacitor’s negative end is connected to the positive plate of previous stage capacitors, the voltage developed across these intermittent capacitors is found to be reduced by a factor of δ_3_. Alternatively, the voltage gain of F-C^3^BC is also derived by applying volt-second balance across inductors *L*_*3*_ to *L*_*5*_ as presented through (15)–(18).

15 $$V_{{L_{{3,ON}} }} \,\,\, + (1 - \delta _{3} )\,(V_{{L_{{3,OFF}} }} ) = \,\,\delta _{3} \,(v_{{C_{1} }} ) + (1 - \delta _{3} )( - v_{{C_{2} }} ) = 0$$

16 $${V_{{L_{4,ON}}}}\,\,\,+(1 - {\delta _3})\,({V_{{L_{4,OFF}}}})=\,\,{\delta _3}\,({v_{{C_1}}}+{v_{{C_2}}})+(1 - {\delta _3})( - v{_{{C_3}}})=0$$

17 $${V_{{L_{5,ON}}}}\,\,\,+(1 - {\delta _3})\,({V_{{L_{5,OFF}}}})=\,\,{\delta _3}\,({v_{{C_1}}}+{v_{{C_2}}}+{v_{{C_3}}})+(1 - {\delta _3})({v_{{C_1}}}+{v_{{C_2}}}+{v_{{C_3}}} - {V_0})=0$$

18 $${M_{Stage2}}=\frac{{{V_o}}}{{{v_{{C_1}}}}}=\,\frac{1}{{{{(1 - {\delta _2})}^3}}}$$

From the voltage gain contributed by the two stages, the overall voltage gain of the Q^4^HGC is obtained and presented in (19).

19 $$M={M_{Stage1}} * {M_{Stage2}}=\frac{{{V_o}}}{{{V_{in}}}}=\,\frac{2}{{(1 - {\delta _1}){{(1 - {\delta _3})}^3}}}$$

**Fig. 4:**
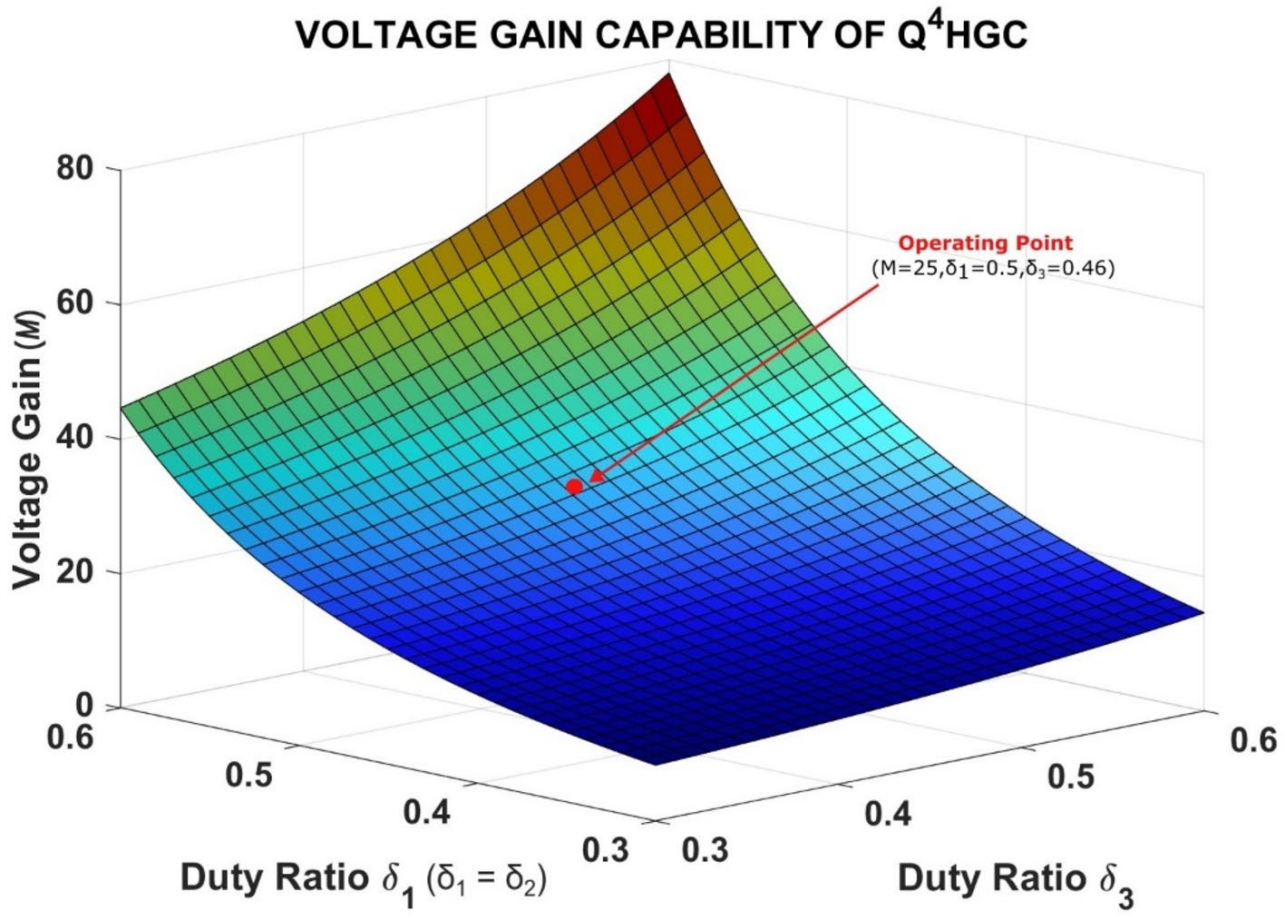
3D plot showing the voltage gain capability of Q^4^HGC and its operating point.

When the duty ratio (*δ*) of both the stages is equal, i.e., *δ*_*1*_ = *δ*_*2*_ = *δ*_*3*_ = *δ*, the effective voltage gain is the 4th power of (quartic times) the voltage gain obtained from a CBC structure. Since the operation of *S*_*3*_ is independent of *S*_*1*_ and *S*_*2*_, the proposed converter possesses two degrees of freedom, viz., its ability to provide the desired voltage amplification while drawing ripple-free current from the source. For instance, *S*_*1*_ and *S*_*2*_ are operated at a fixed duty ratio of 0.5 with a phase shift of 180° to eliminate the input current ripple while *δ*_*3*_ is adjusted to obtain the required voltage gain. Thus, the proposed converter is well-suited for PV applications as its duty ratio values are safe and the input current is also free from ripples. Figure 4 exhibits the voltage gain capability of the proposed Q^4^HGC along with its practical operating point.

### Switch ratings

In the proposed converter, since *S*_*1*_ and *S*_*2*_ are part of the IBC structure, they experience the same voltage stress as that of the CBC and is given by (20).

20 $${V_{{S_1}}}={V_{{S_2}}}=\,\frac{{{V_{in}}}}{{(1 - {\delta _1})}}\,\,=\frac{{{{(1 - {\delta _1})}^3}\,{V_0}}}{2}$$

The current stress of *S*_*1*_ is determined when it is conducting (during mode 1 or mode 2). As *L*_*1*_ charges through *S*_*1*_ and the input current is shared by the two interleaved phases, the current stress of *S*_*1*_ is given by (21).

21 $${I_{{S_1}}}=\,{I_{{L_1}}}\,\,=\frac{{{I_{in}}}}{2}$$

When *S*_*2*_ is turned ON, *L*_*1*_ transfers its energy to *C*_*Lift*_ through *S*_*2*_ while *L*_*2*_ also charges through *S*_*2*_. Hence, its current stress is relatively higher than that of *S*_*1*_, and itd is given by (22).

22 $${I_{{S_2}}}=\,{I_{{L_1}}}+{I_{{L_2}}}\,\,={I_{in}}$$

Since *S*_*3*_ is located near the output port, its voltage stress is the same as *V*_*0*_. When *S*_*3*_ is ON, inductors *L*_*3*_, *L*_*4*_, and *L*_*5*_ charge through *S*_*3*_. Hence, its current stress is given by the sum of current through inductors *L*_*3*_, *L*_*4*_, and *L*_*5*_ as expressed in (23).

23 $${I_{{S_3}}}=\,{I_{{L_3}}}+{I_{{L_4}}}+\,{I_{{L_5}}}\,={I_{in}}\frac{{(1 - {\delta _1})((2 - {\delta _2})+{{(1 - {\delta _2})}^2})}}{2}$$

### Ratings of diodes

The diodes experience voltage stress when operating in reverse-biased conditions. Their voltage stress magnitudes are quantified as the potential difference across their anode and cathode terminals. *D*_*1*_ is reverse-biased during mode 1; its anode is clamped to the ground by *S*_*1*_, and its cathode is connected to *C*_*1*_. Hence, its voltage stress is given by (24). Similarly, *D*_*2*_ is reverse-biased when *S*_*2*_ is turned ON during mode 3. Its anode and cathode terminals are connected to *C*_*Lift*_ and *C*_*1*_ respectively. Therefore, it experiences a voltage stress that is given by (25).

24 $${V_D}_{{_{1}}}=\,{v_{{C_1}}}\,\,=\frac{{2{V_{in}}}}{{(1 - {\delta _1})}}={V_0}{(1 - {\delta _3})^3}$$

25 $${V_D}_{{_{2}}}=\,{v_{{C_1}}} - {v_{{C_{Lift}}}}\,\,=\frac{{{V_0}(1 - {\delta _3})}}{2}$$

Diodes *D*_*1*_ and *D*_*2*_ carry the currents that flow through *L*_*1*_ and *L*_*2*_, respectively. Hence, their current stress level is given by (26).

26 $${I_D}_{{_{1}}}=\,{I_{{L_1}}},{I_D}_{{_{2}}}=\,{I_{{L_2}}}\,\,=\frac{{{I_{in}}}}{2}$$

Diodes *D*_*3*_ and *D*_*5*_ are reverse-biased during modes 2 and 3. The anode and cathode terminals of *D*_*3*_ are connected to *C*_*2*_ and *C*_*3*_, respectively. Hence its voltage stress is given by (27). In the case of *D*_*5*_, its cathode terminal is clamped at *V*_*0*_, and its anode is at a relatively lower potential level of *C*_*3*_; its voltage rating is given by (28).

27 $${V_D}_{{_{3}}}=\,{v_{{c_3}}}\,\,=\frac{{2{\delta _3}{V_{in}}}}{{(1 - {\delta _1}){{(1 - {\delta _3})}^2}}}={\delta _3}{V_0}(1 - {\delta _3})$$

28 $${V_D}_{{_{5}}}=\,{V_0} - {v_{{c_1}}} - {v_{{c_2}}} - {v_{{c_3}}}\,\,=\frac{{2{\delta _3}{V_{in}}}}{{(1 - {\delta _1}){{(1 - {\delta _3})}^3}}}={\delta _3}{V_0}$$

During mode 1, the diodes *D*_*3*_ and *D*_*5*_ are forward-biased and carry the currents that flow through *L*_*3*_ and *L*_*4*_, respectively. Therefore, their current stress is given by (29) and (30).

29 $${I_D}_{{_{3}}}=\,{I_{{L_3}}}\,\,=\frac{{(1 - {\delta _1}){I_{in}}}}{2}$$

30 $${I_D}_{{_{5}}}=\,{I_{{L_4}}}\,\,=\frac{{(1 - {\delta _1})(1 - {\delta _3}){I_{in}}}}{2}$$

During mode 1, diodes *D*_*4*_ and *D*_*6*_ are under reverse-biased conditions. Consequently, the anode of *D*_*4*_ is grounded as switch *S*_*3*_ conducts. Hence, its voltage stress is given by determining the voltage developed across *C*_*3*_ with respect to ground and expressed using (31). Similarly, *D*_*6*_ experiences a voltage stress value that is equal to the sum of potentials developed across the capacitors *C*_*1*_, *C*_*2*_, and *C*_*3*_ as expressed in (32).

31 $${V_D}_{{_{4}}}=\,{v_{{c_2}}}+{v_{{c_1}}}\,\,=\frac{{2{V_{in}}}}{{(1 - {\delta _1})(1 - {\delta _3})}}={V_0}{(1 - {\delta _3})^2}$$

32 $${V_D}_{{_{6}}}=\,{v_{{c_1}}}+{v_{{c_2}}}+{v_{{c_3}}}\,\,=\frac{{2{V_{in}}}}{{(1 - {\delta _1}){{(1 - {\delta _3})}^2}}}={V_0}{(1 - {\delta _3})^3}$$

The diodes *D*_*4*_ and *D*_*6*_ operate in mode 2 or 3 when *S*_*3*_ is turned OFF and carry the current through *L*_*3*_ and *L*_*4*_, respectively. Therefore, their current stress is expressed by (33) and (34). Since *D*_*7*_ is located close to the output port, its voltage stress is equal to *V*_*0*_, and it carries current through inductor *L*_*4*_.

33 $${I_D}_{{_{4}}}=\,{I_{{L_3}}}\,\,=\frac{{(1 - {\delta _1}){I_{in}}}}{2}$$

34 $${I_D}_{{_{6}}}=\,{I_{{L_4}}}\,\,=\frac{{(1 - {\delta _1})(1 - {\delta _3}){I_{in}}}}{2}$$

### Design of magnetics

The optimal characteristics of the magnetic elements play a crucial role in ensuring the effective operation of the power converter. The inductor values in boost-derived converters are derived based on the duty ratio (δ), voltage across the individual inductors, operating frequency, and the individual current ripple values. The generalized equation to arrive at the inductor value is given by (35).

35 $${L_x}=\,\frac{{{\delta _i}\,{V_{{L_x},ON}}}}{{{f_s}\Delta {i_{{L_x}}}}},{\text{ }}i{\text{=1 to 3, }}x=1{\text{ to }}5$$

where *δ* is the duty ratio of the switch, ${V_{{L_x},ON}}$is the voltage across the individual inductor, $\Delta {i_{{L_x}}}$is the individual inductor current ripple, and *f*_*s*_ is the switching frequency.

For CCM operation, the actual value of the inductors must be more than the critical inductance (*L*_*critical*_) value that is expressed using (35a).

35a $${L_{x - critical}}=\,\frac{{\delta {R_0}}}{{2{f_s}{M^2}}},{\text{ }}x={\text{1 to 5}}$$

where *R*_*0*_ is the load resistance value.

In the proposed converter, *L*_*1*_ and *L*_*2*_ operate under identical conditions. Hence, their value is determined using (36) and (37).

36 $${L_1}=\,\frac{{{\delta _1}\,{V_{in}}}}{{{f_s}\Delta {i_{{L_1}}}}}\,$$

37 $${L_2}=\,\frac{{{\delta _2}\,{V_{in}}}}{{{f_s}\Delta {i_{{L_2}}}}}$$

Inductor *L*_*3*_ is charged by the capacitor *C*_*1*_ when *S*_*3*_ is ON. Therefore, the value of *L*_*3*_ is determined using (38).

38 $${L_3}=\,\frac{{{\delta _3}\,{v_{{c_1}}}}}{{{f_s}\Delta {i_{{L_3}}}}}=\frac{{2{\delta _3}{V_{in}}}}{{{f_s}\Delta {i_{{L_3}}}(1 - {\delta _1})}}$$

The voltage impressed across *L*_*4*_ and *L*_*5*_ is considerably higher since they are charged by the series combination of the previous stage capacitors. Hence, their values are obtained using the design expressions presented in (39) and (40).

39 $${L_4}=\,\frac{{{\delta _3}\,({v_{{c_1}}}+{v_{{c_2}}})}}{{{f_s}\Delta {i_{{L_4}}}}}=\frac{{2{\delta _3}{V_{in}}}}{{{f_s}\Delta {i_{{L_4}}}(1 - {\delta _1})(1 - {\delta _3})}}$$

40 $${L_5}=\,\frac{{{\delta _3}\,({v_{{c_1}}}+{v_{{c_2}}}+{v_{{c_3}}})}}{{{f_s}\Delta {i_{{L_5}}}}}=\frac{{2{\delta _3}{V_{in}}}}{{{f_s}\Delta {i_{{L_5}}}(1 - {\delta _1}){{(1 - {\delta _3})}^2}}}$$

### Determination of capacitance values

The capacitance values are determined based on the ripple voltage impressed across the individual capacitors and the current that passes through them. The capacitance value of *C*_*1*_ and *C*_*2*_, which is in stage 1, is given by (41).

41 $${C_{1,2}}=\,\frac{{{\delta _{1,2}}{I_{in}}}}{{{f_s}\Delta {v_{{C_{1,2}}}}}}$$

In the proposed Q^4^HGC, capacitors in stage 1 have higher capacitance values as they carry higher currents. The capacitors in stage 2 carry lower currents due to the voltage gain.

**Table 1:** Specifications of the proposed converter.

Parameter	Value
Input voltage (*V*_*in*_)	16 V
Switching frequency (*f*_*s*_)	100 kHz
Duty ratio of *S*_*1*_, *S*_*2*_ (*δ*_*1*_)	0.5
Output voltage (*V*_*0*_)	400 V
Duty ratio of *S*_*3*_ (*δ*_*3*_)	0.46
Output power (*P*_*0*_)	150 W

**Table 2:** Components used to fabricate and test the prototype Q^4^HGC.

Circuit element	Device type	Part number (specifications)
Switch (*S*_*1*_, *S*_*2*_)	MOSFET	IRFB4410Z (100 V, 88 A, 8 mΩ)
Switch (*S*_*3*_)	MOSFET	NTHL041N60S5H (600 V, 57 A, 33 mΩ)
Diodes (*D*_*2*,_*D*_*1*_)	Fast recovery diode	DSS 16–01 A (100 V, 16 A, 0.64 V)
Diodes (*D*_*2*,_*D*_*4*_, *D*_*5*,_*D*_*6*_)	Fast recovery diode	MUR1540 (400 V,15 A, 1.05 V)
Diode (*D*_*3*_)	Fast recovery diode	MUR1520 (200 V, 15 A, 0.85 V)
Diode (*D*_*7*_)	Fast recovery diode	BYV29X-600 (600 V, 9 A, 1.26 V)
Inductors (*L*_*1*_, *L*_*2*_, *L*_*3*_)	Ferrite core	PCV2-104–10 L (100 µH, 10 A)
Inductor (*L*_*4*_)	Ferrite core	PCV2-564–6 L (564 µH, 6 A)
Inductor (*L*_*5*_)	Ferrite core	PCV2-105–02 L (1 mH, 2 A)
Capacitors (*C*_*lift*_, *C*_*2*_, *C*_*3*,_*C*_*4*_*)*	Polypropylene	Film Capacitor (3.3 µF, 250 V)
Capacitor (*C*_*0*_)	Electrolytic	B43644J65 66M067 (56 µF, 500 V)
Capacitor (*C*_*1*_)	Electrolytic	EKMG101ELL101MJ20S (100 µF, 100 V)

Their capacitance values are obtained from the expression given by (42).

42 $${C_k}=\,\frac{{{\delta _3}{I_{{C_k}}}}}{{{f_s}\Delta {v_{{C_k}}}}},{\text{ }}k=3,4,5$$

## Experimental results and discussion

**Fig. 5:**
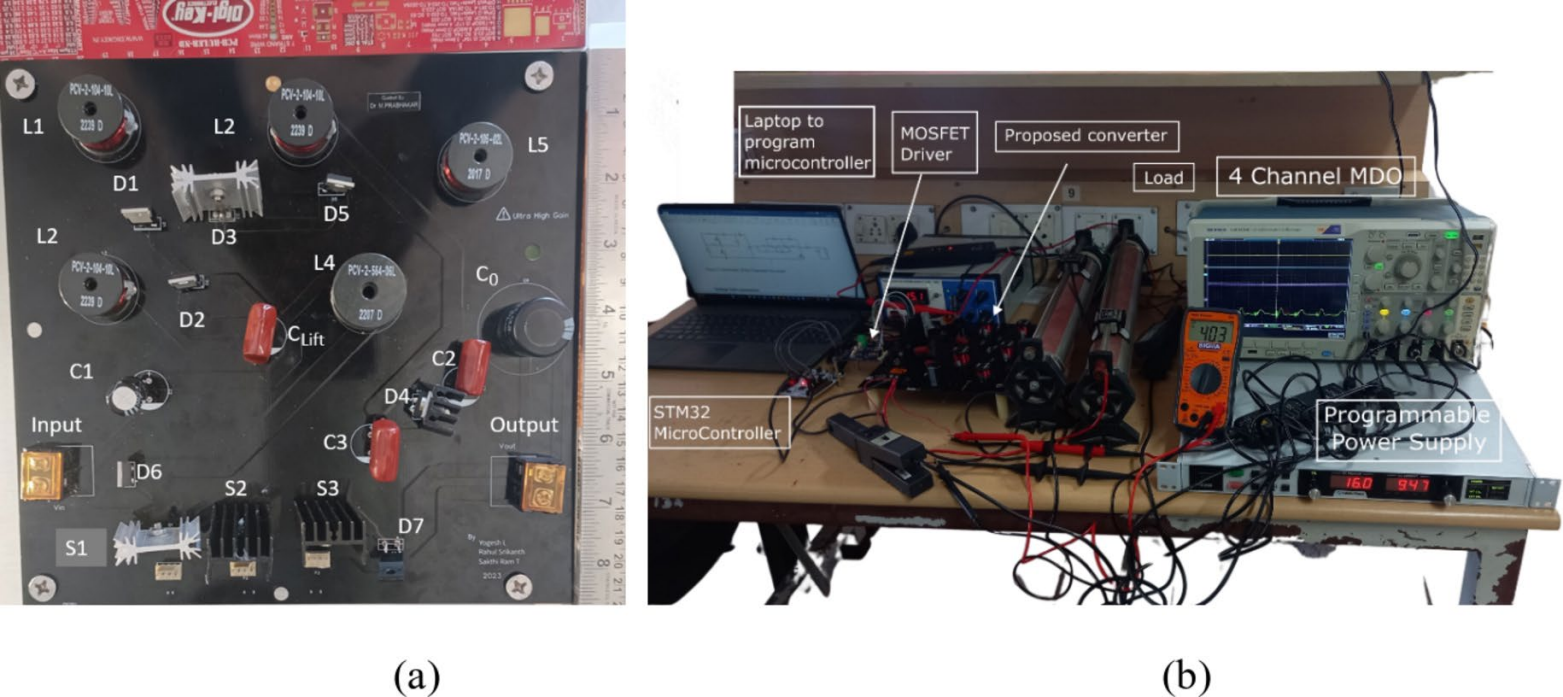
Photograph showing (**a**) the top-view of the prototype Q^4^HGC, and (**b**) the experimental setup used to capture the results.

To validate the proposed voltage gain hypothesis, a laboratory prototype model of the Q^4^HGC is fabricated with the specifications mentioned in Table 1. The components listed in Table 2 are used to construct the prototype converter. An Arm Cortex^®^-M4-based processor STM32F411RE Nucleo-64 microcontroller is used to generate the gating signal and implement the closed-loop control algorithm. The gate pulses generated from the microcontroller are applied to IR25600 dual low-side MOSFET drivers. The amplified pulses are applied to the gate terminal of the power switches. The key waveforms obtained from the prototype converter are captured using a mixed-domain oscilloscope (MDO4014C) along with differential high-voltage and current probes. Figure 5(a) depicts the photograph of the prototype Q^4^HGC. The experimental setup that is used to obtain the test results is portrayed in Fig. 5(b).

**Fig. 6:**
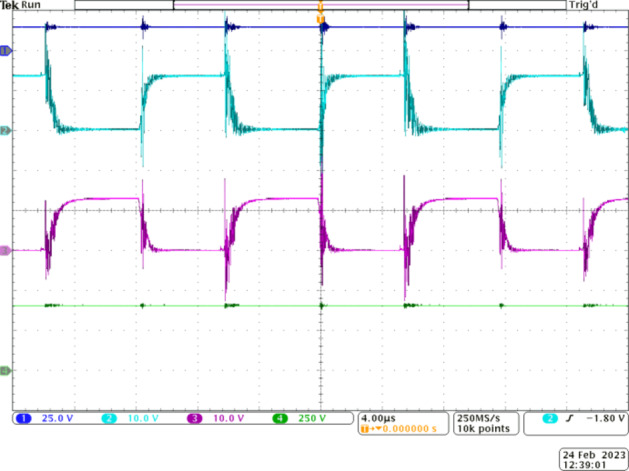
Experimental results to demonstrate the voltage gain capability of the proposed Q^4^HGC. CH1—input voltage, CH2—gate pulse to *S*_*1*_, CH3—gate pulse to *S*_*2*_, and CH4—output voltage.

Figure 6 depicts the voltage gain capability of the proposed Q^4^HGC. The gating pulses are applied to *S*_*1*_ and *S*_*2*_ (CH2 and CH3) at a duty ratio of 0.5 with a 180° phase-shift. CH1 depicts the input voltage (16 V), and the voltage developed across the output is shown in CH4. Obviously, 400 V is obtained across the output terminals when the input is 16 V. Thus, the proposed voltage gain concept is validated; the practical voltage gain value is 25.

**Fig. 7:**
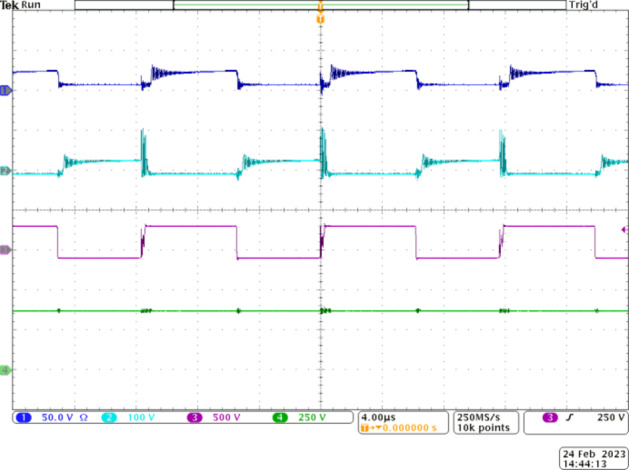
Experimental waveforms to validate the voltage stress across the switches of the proposed Q^4^HGC. CH1, CH2, CH3 –voltage across *S*_*1*_, *S*_*2*_, and *S*_*3*_ respectively, CH4—output voltage.

Figure 7 validates the proper operation of the switches and their voltage stress levels with respect to the output voltage. CH1 and CH2 show the voltage across switch *S*_*1*_ and *S*_*2*_, respectively. Their complementary operation is verified from these oscillograms. *S*_*1*_ and *S*_*2*_ share similar voltage stress due to their complementary operation. Additionally, since they are employed in stage 1, their voltage stress is practically 32 V, which is only 8% of *V*_*0*_.

**Fig. 8:**
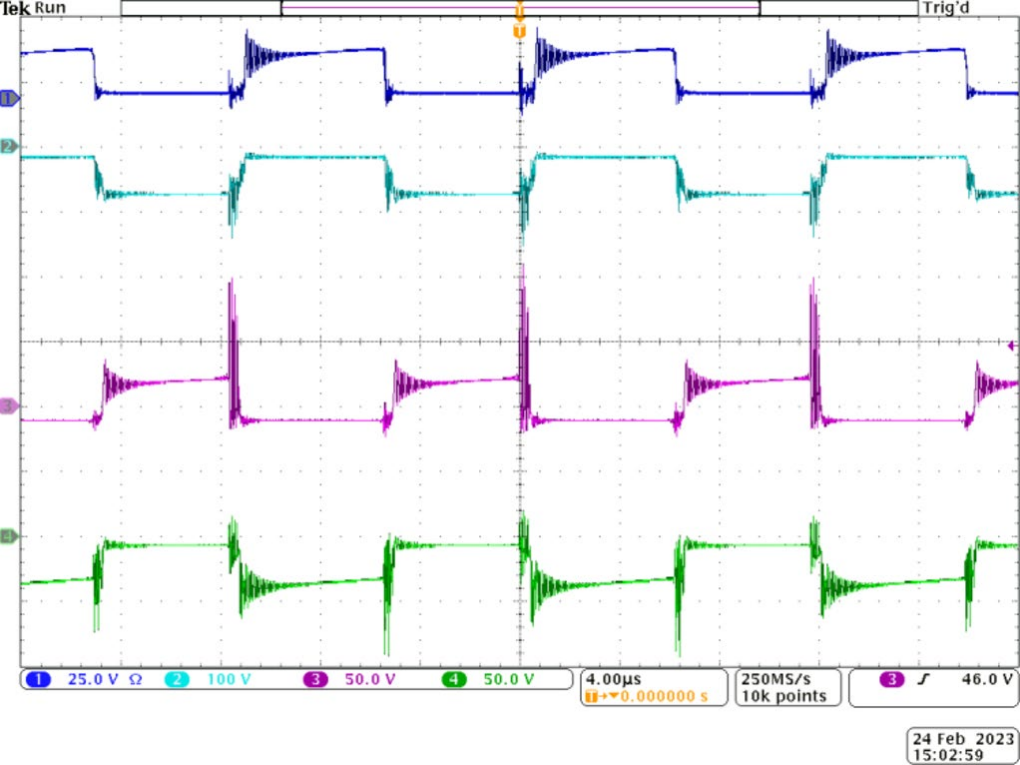
Experimental waveforms to demonstrate the operation of IBC stage, CH1—voltage across *S*_*1*_, CH2—voltage across *D*_*1*_, CH3—voltage across *S*_*2*_, and CH4—voltage across *D*_*2*_.

**Fig. 9:**
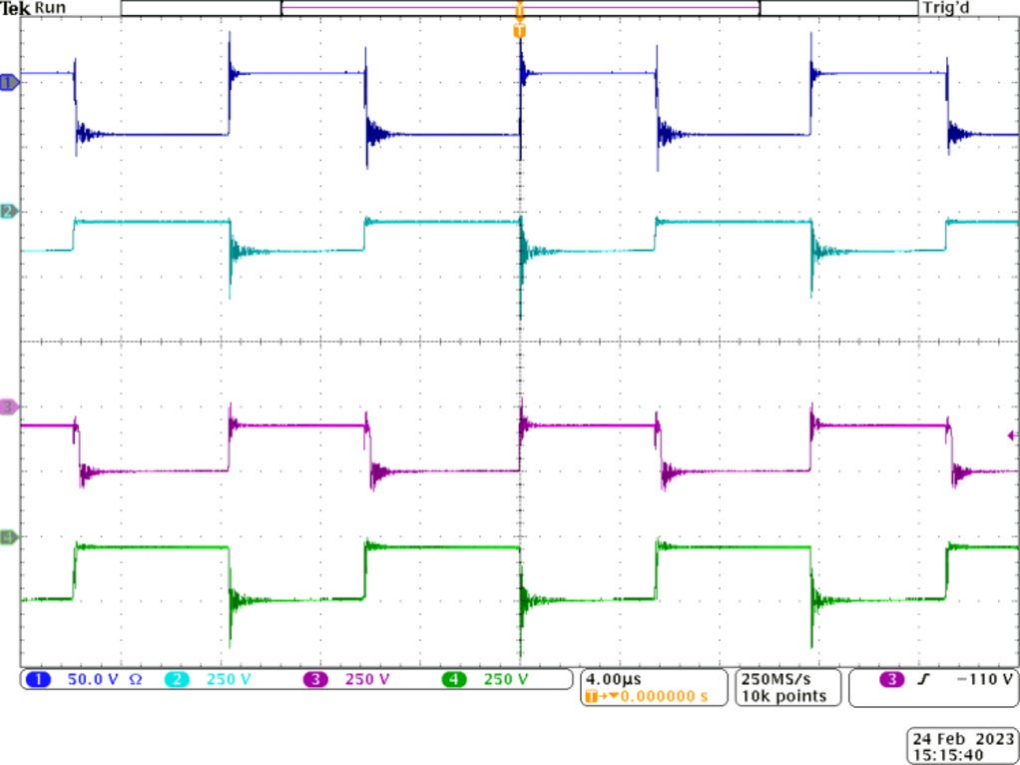
Experimental waveforms to demonstrate the working of stage 2. CH1—voltage across *D*_*1*_, CH2—voltage across *D*_*4*_, CH3—voltage across *D*_*5*_, and CH4—voltage across *D*_*6*_.

The practical value matches with the analytical value. Since the voltage stress on the two switches is very low, MOSFETs with very low *R*_*DS−ON*_ are employed to lower the conduction losses. The operation of *S*_*3*_ is synchronized with *S*_*1*_. The switch *S*_*3*_ is operated at a slightly lower duty ratio of 0.46 and is adjusted to achieve the required voltage gain at nominal load conditions. Since *S*_*3*_ is located closer to the output port, its voltage stress is *V*_*0*_ as depicted in CH3. However, since the current stress on *S*_*3*_ is reduced, its loss is reduced.

Figure 8 demonstrates the complementary operation of *S*_*1*_ and *D*_1_ through the waveforms portrayed in CH1 and CH2. Similarly, CH3 and CH4 validate the complementary working *S*_*2*_ and *D*_*2*_ as per the operating principle. Further, diode *D*_*1*_ experiences a voltage stress of around 64 V (16% of *V*_*0*_) as observed from CH2. The value matches with the theoretically calculated counterpart. From the CH4 waveform, *D*_*2*_ experiences a voltage stress of about 32 V, which is the same as that of *S*_*1*_ and *S*_*2*_. Therefore, all switching elements in stage 1, except *D*_1_, experience a very low voltage stress of 32 V, which is just 8% of *V*_*0*_, while the voltage stress on *D*_*1*_ is 16% of *V*_*0*_. Once again, the lower voltage stress on the diodes employed in stage 1 permits the choice of diodes with lower voltage drops to enhance the efficiency.

Figure 9 demonstrates the operation of stage 2. The voltage stress across the diodes *D*_*1*_, *D*_*4*_, *D*_*5*_, and *D*_*6*_ is depicted through the waveforms in CH1 to CH4, respectively. Diode *D*_*5*_ is expected to operate when *S*_*3*_ is ON. Hence, *D*_*5*_ and *S*_*3*_ operate symmetrically and is verified from CH3 and CH4. Diode *D*_*5*_ experiences a higher voltage stress of 185 V as its cathode terminal is clamped to *V*_*0*_ when *S*_*3*_ is OFF. Diodes *D*_*4*_ and *D*_*6*_ operate complementary to *D*_*3*_ and *D*_*5*_ respectively. Diode *D*_*4*_ experiences a voltage stress of 121 V, which accounts for only 30% of *V*_*0*_ while *D*_*6*_ is subjected to a higher voltage stress of 221 V, which is in accordance with the theoretical calculations. Except for *D*_*6*_ and *D*_*7*_, the voltage stress on the remaining diodes used in the floating capacitor cell is well below 50% of *V*_*0*_. Thus, the choice of an appropriate gain extension mechanism is validated.

**Fig. 10:**
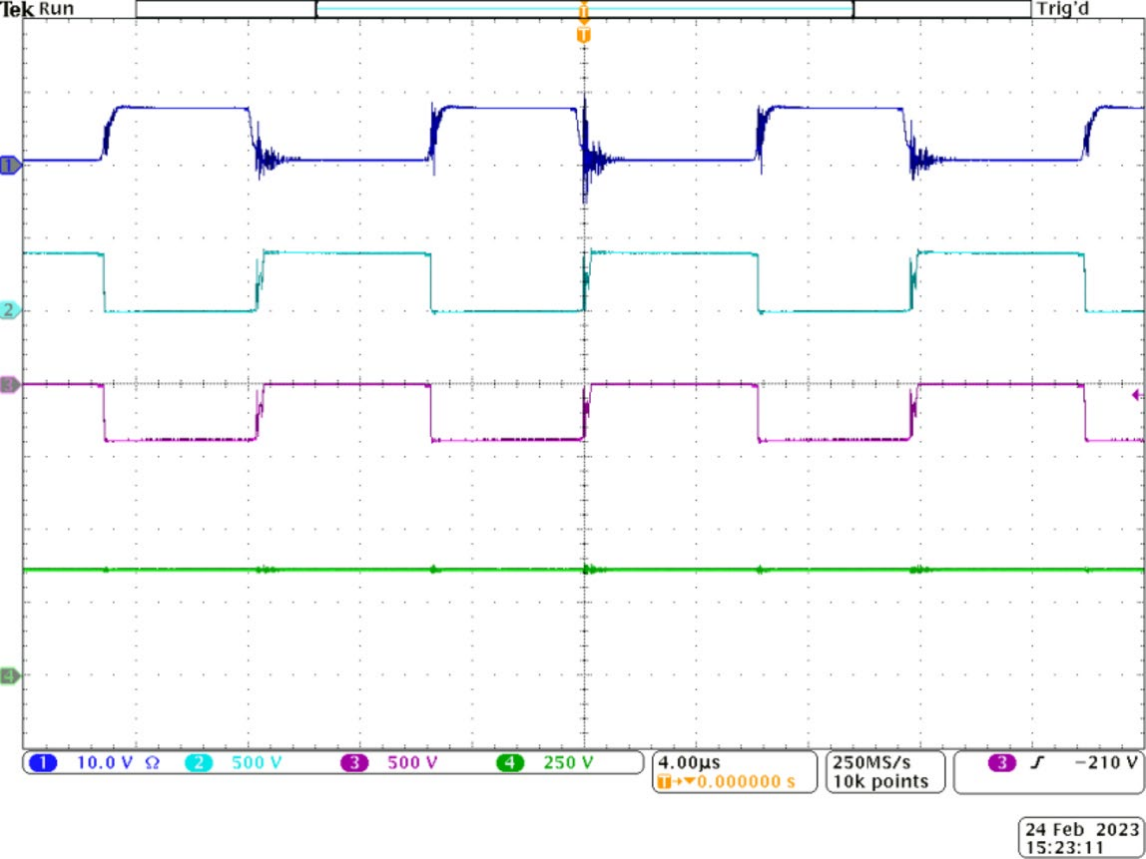
Experimental waveforms to demonstrate the operation of *S*_*3*_ and *D*_*7*_. CH1—gate pulses to *S*_*3*_, CH2—voltage stress on *S*_*3*_, CH3—voltage stress on *D*_*7*_, and CH4—*V*_*0*_.

The correlated operation of *S*_*3*_ and *D*_*7*_ is verified through the experimental waveforms presented in Fig. 10. Switch *S*_*3*_ is operated at a very low duty ratio of 0.46 as observed from CH1. The voltage stress across *S*_*3*_ is portrayed through CH2, and the value is as expected. Since *S*_*3*_ is located closer to the output terminals, its voltage stress is the same as *V*_*0*_. The complementary operation of *D*_*7*_ with respect to *S*_*3*_ is also demonstrated by correlating the waveforms presented in CH2 and CH3. Diode *D*_*7*_ is also subjected to a voltage stress that is equal to *V*_*0*_ based on its location. However, the switching and conduction losses across *S*_*3*_ and *D*_*7*_ are significantly reduced due to their lower current stress levels.

**Fig. 11:**
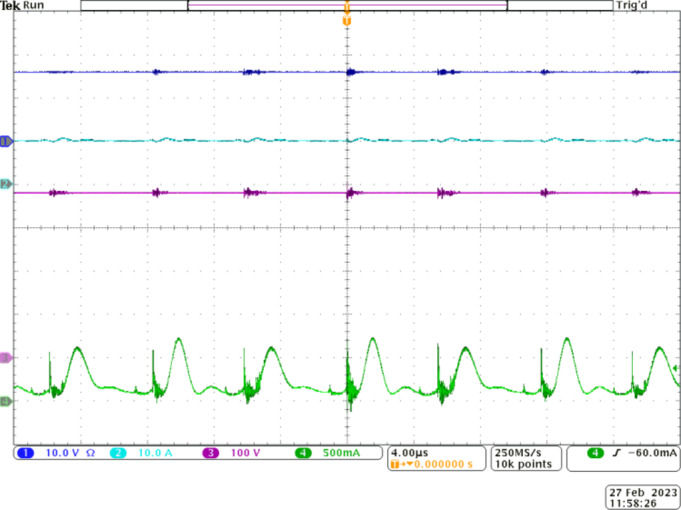
Experimental waveforms to demonstrate the efficiency of the proposed Q^4^HGC at full-load condition. CH1—*V*_*in*_, CH2—input current, CH3—*V*_*0*_, and CH4—load current.

Figure 11 depicts the efficiency of the proposed converter under full-load conditions. From the waveforms, the converter delivers 150 W to the load at an output voltage of 400 V. The input current is smooth and almost ripple-free due to the interleaving mechanism employed in stage 1.

**Fig. 12:**
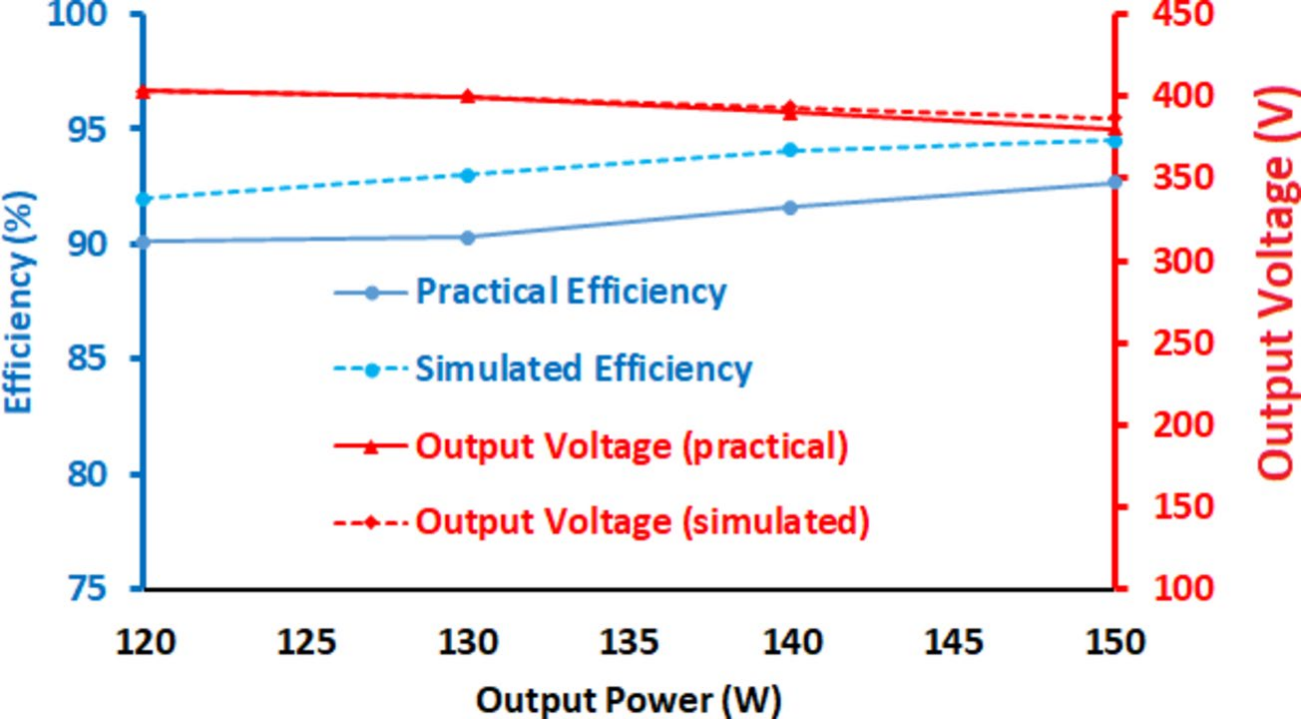
Efficiency curve at various load conditions during simulation and experimentation.

**Fig. 13:**
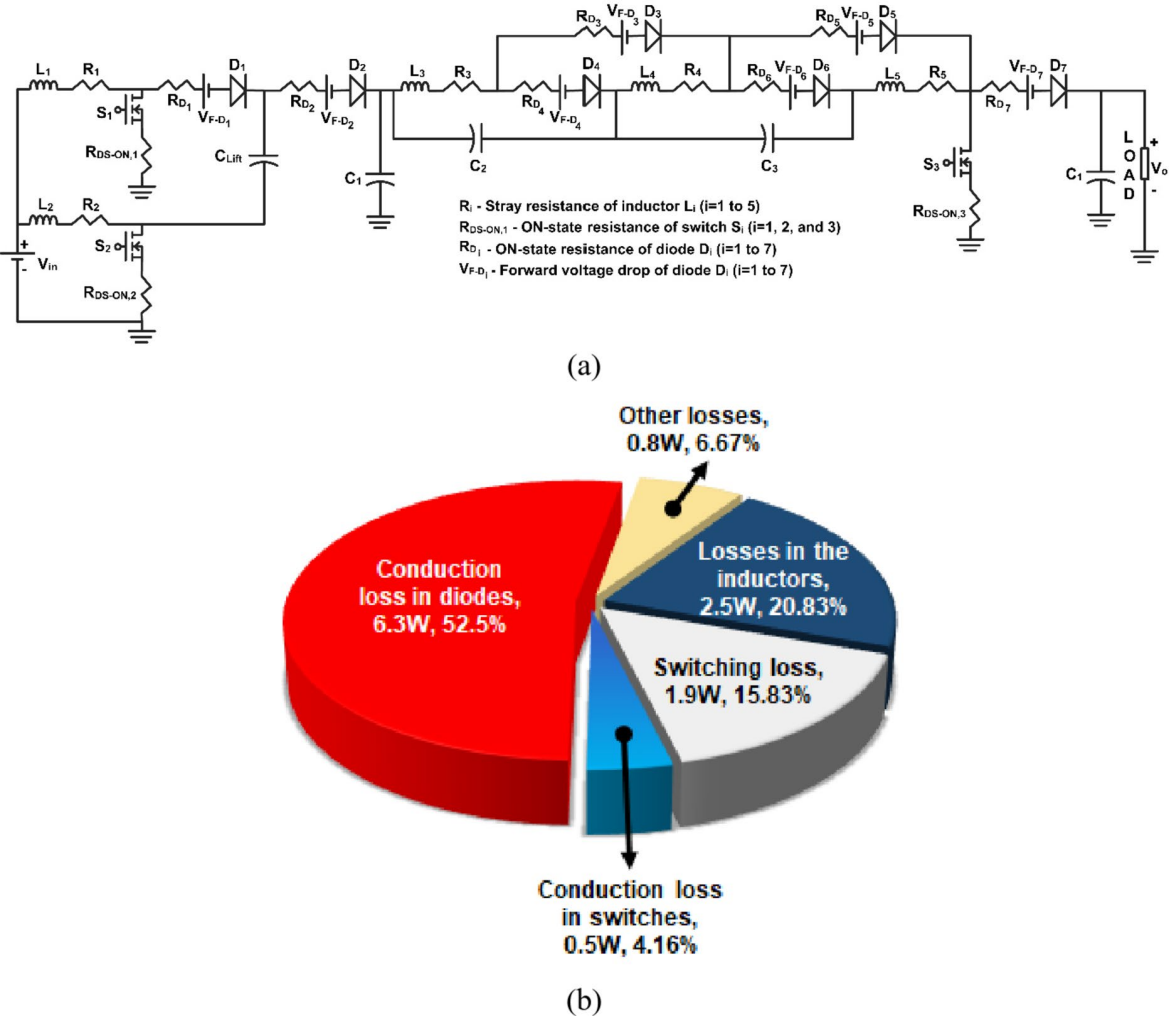
Diagrams depicting the (**a**) equivalent circuit with non-ideal elements and (**b**) loss distribution profile of the Q^4^HGC under full-load condition.

The magnitude of the input current is computed to be 10.11 A when 16 V is applied. Thus, the prototype converter operates at an impressive efficiency of 92.7% under full-load conditions. The efficiency plot of the proposed Q^4^HGC at different load conditions is portrayed in Fig. 12. The practical values match closely with the simulated values; the minor variation in simulation is due to the stray losses associated with the passive elements.

The non-ideal elements of the power circuit are (i) the stray resistance of the inductors, (ii) ON-state resistance of the switches, (iii) the ON-state resistance of the diodes, and (iv) the ON-state voltage drop of the diodes. Figure 13(a) shows the equivalent circuit of the proposed power converter with all the non-ideal elements. The power loss dissipated across the various components of the proposed converter is categorized as (i) loss across the switch, (ii) loss across the diodes, and (iii) loss across the inductors^9,10,39^. Based on the equations presented in (43)-(47), the power loss distribution of the proposed converter at full-load condition is obtained and plotted in Fig. 13(b). Most of the losses occur across the diodes.

43 $${P_{{\text{sw,loss}}}}={I^2}_{{{\text{sw,RMS}}}} \times {R_{{\text{sw,ON}}}}+{P_{{\text{sw,ON}}}}+{P_{{\text{sw,OFF}}}}$$

44 $${I_{{\text{sw,RMS}}}}=\sqrt {\frac{D}{3}} {I_{{\text{sw}}}}$$

45 $${P_{diode\_loss}}=\left( {{V_{diode\_ON}} \times {I_{diode}}} \right)+\left( {I_{{diode}}^{2} \times {R_{diode\_ON}}} \right)$$

46 $${I_{{\text{diode,RMS}}}}=\sqrt {\frac{D}{3}} {I_{diode}}$$

47 $${P_{inductor\_loss}}={I^2}_{{inductor}}{R_{inductor}}+{P_{iron}}$$

**Fig. 14:**
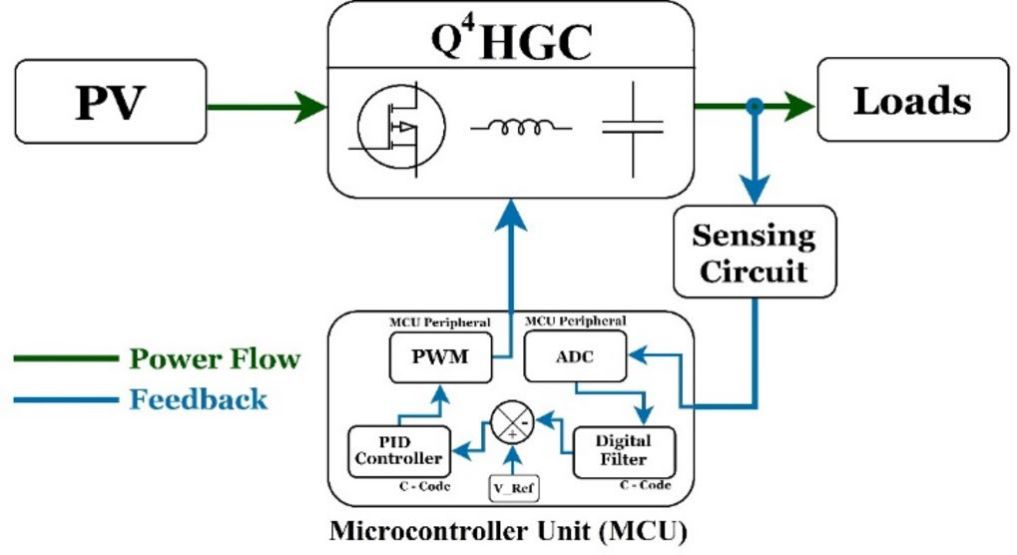
Block diagram of the closed-loop control implementation.

The dynamic performance of the proposed converter is examined by implementing a simple closed-loop control technique. A potential divider-based network is adopted to reduce the actual output voltage to a safer level (i.e., < 3.3 V) and fed as input to the analog-to-digital converter (ADC) peripheral of the microcontroller. The output of the ADC is infested with noises and is filtered by implementing an appropriate software-based filter. The filtered-ADC output is used to obtain the error signal by comparing it with the reference voltage. The error signal is then fed to a discrete-time proportional controller with lower and upper saturation values on duty ratio to protect the switches. The controller is suitably tuned to provide a very quick response with minimal overshoot. Figure 14 depicts the schematic block diagram of the closed-loop control technique that is employed.

**Fig. 15:**
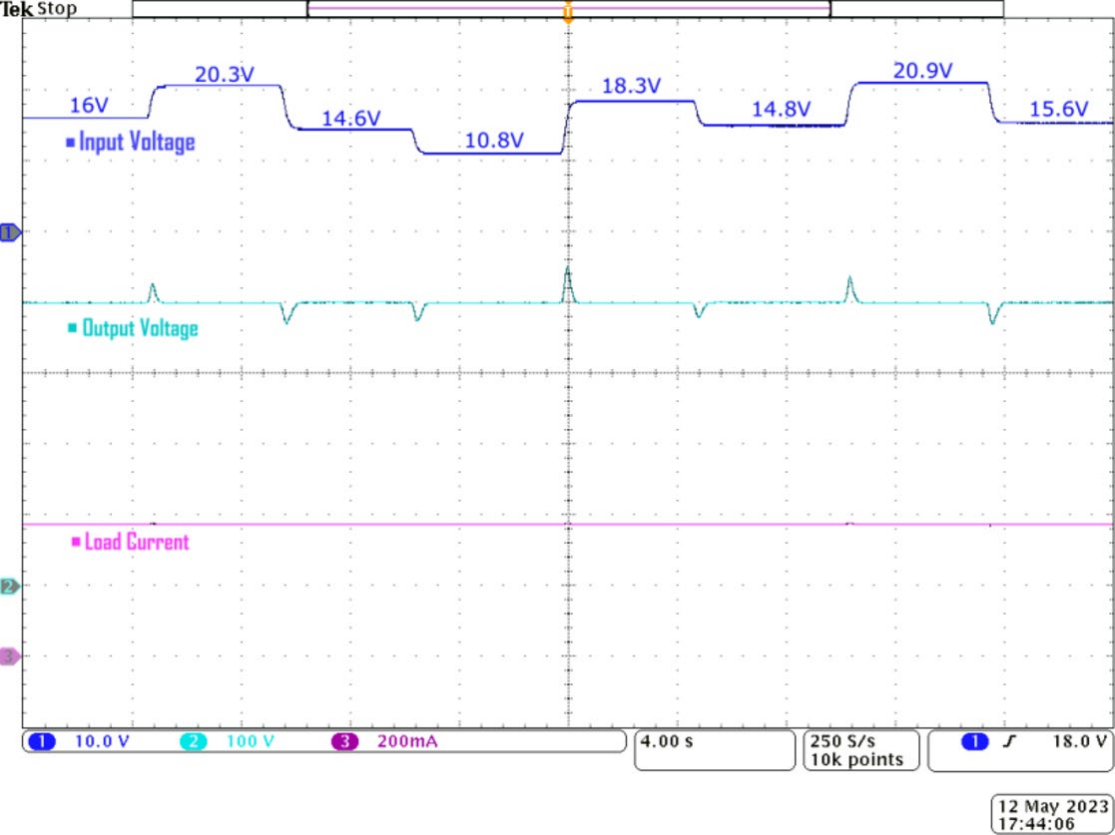
Experimental waveforms to showcase the line voltage regulation capability of the proposed Q^4^HGC, CH1—input voltage, CH2—output voltage, and CH3—load current.

Figure 15 demonstrates the line regulation characteristics of the prototype converter. Under nominal conditions, the Q^4^HGC delivers 150 W to the load at 400 V. The duty ratio of *S*_*3*_ is adjusted by using closed-loop control. When the input voltage is randomly varied from 10.8 to 20.9 V in a stepped manner, the closed-loop control mechanism acts on the converter, and the output voltage value is quickly restored to the nominal value of 400 V with minimal undershoot and overshoot. The proposed converter momentarily achieves a maximum voltage amplification of 37 while stepping up from 10.8 to 400 V.

**Fig. 16:**
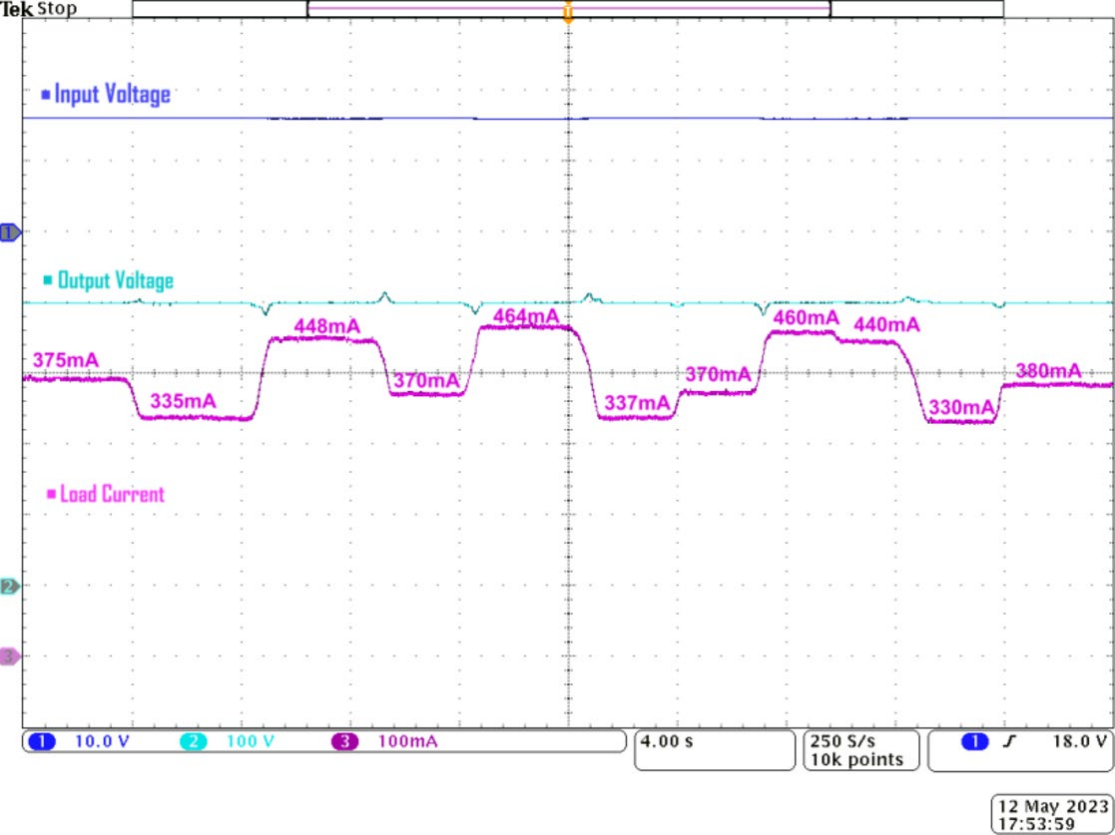
Experimental waveforms to verify the load regulation capability of the proposed converter, CH1—input voltage, CH2—output voltage, and CH3—load current.

Figure 16 illustrates the load regulation capability of the proposed Q^4^HGC. The proposed converter effectively maintains a stable output voltage across a broad spectrum of load current changes, and is evident from the practical waveforms. The proposed Q^4^HGC meets the load requirements at 400 V even when the load current varies from 330 to 464 mA. In terms of power levels, the proposed converter maintains the output voltage at 400 V when the load fluctuates between 132 and 185 W. This practical evidence confirms the converter’s adaptability and reliability in managing the diverse load scenarios despite line voltage fluctuations while delivering a constant voltage of 400 V to the output.

## Benchmarking the proposed converter

In this section, the proposed converter is compared with recent and similar state-of-the-art high-gain DC–DC converters to appreciate its superior features. The converters that are chosen for comparison are presented in various references as outlined in Table 3. To understand and validate the superior features of the proposed Q^4^HGC, the converters that are chosen for comparison yield a voltage gain > 13 and belong to either quadratic, cubic, or quartic variants. The main comparison attributes are elaborated in the following sub-sections. All the converters compared provide voltage amplification greater than 10.

### Voltage gain (*M*) and duty ratio

All the converters that are compared in Table III yield excellent voltage conversion ratios. The converter presented in^36^ yields the maximum voltage gain of 32.5 at a slightly higher duty ratio of 63%. Converters presented in^31,34^ attain the second lowest voltage amplification of 14.8 and 14.16, respectively. The converter in^31^ is a QBC variant that is operated at a duty ratio of 50%, while the converter in^34^ yields twice the cubic amplification using a VMC-based single switch C^3^BC. Moreover, the converter in^34^ is operated at the second lowest duty ratio of 48%, resulting in the second lowest voltage gain value. The converter introduced in^39^ and the proposed Q^4^HGC utilize an interleaved arrangement at the input side to mitigate input current ripple.

**Fig. 17:**
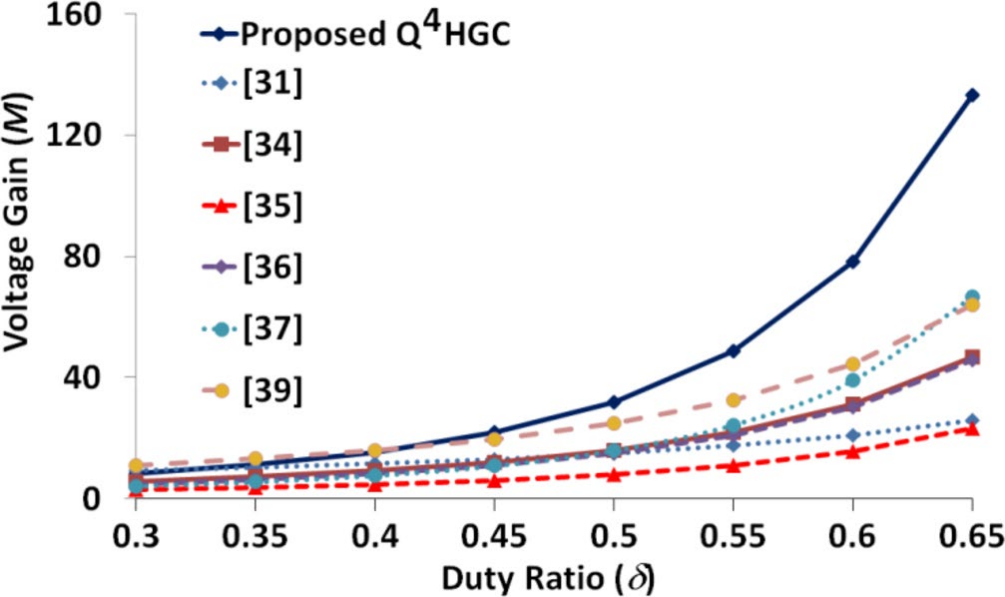
Voltage gain plot of all the converters compared in Table 3.

The proposed Q^4^HGC achieves the second-highest voltage amplification of 25, albeit operating *S*_*3*_ at a notably low duty ratio of 46%, the lowest among all compared converters. On the other hand, the converter outlined in^39^ achieves an impressive voltage amplification of 21.11, operating *S*_*3*_ at a much higher duty ratio of 57%. The converter presented in^37^ yields the lowest voltage conversion ratio of 13.5 at the second lowest duty ratio of 48%. The converter described in^36^ attains the highest voltage amplification, albeit at the second highest duty ratio value of 63%. Figure 17 illustrates the voltage gain plot of all the converters compared in Table 3. The proposed Q^4^HGC yields the highest voltage conversion ratio mainly due to the gain extension techniques adopted.

### Total components used (*TCU*) and *M/TCU* ratio

To gain a deeper understanding of the components used in the converter, the ratio of voltage gain (*M*) to total components used (*TCU*) serves as a comparative metric. The excellent voltage gain values provided by all converters are understood from the *M/TCU* value, which exceeds 1 for all the converters except the converters presented in^34,37^. Despite possessing excellent voltage gain profiles, their performance is hindered primarily due to the lower duty ratio of 48%. The converter outlined in^36^ demonstrates an exceptional *M/TCU* ratio of 2.03, which is the highest. The second highest *M/TCU* value is 1.48, which is obtained by the converter in^35^. The converters in^35,36^ utilize only 12 and 16 components, respectively. However, as both these converters were operated at significantly higher duty ratio values, they yielded higher voltage gain values.

The converter presented in^39^ achieves a remarkable *M/TCU* of 1.31 at a much lower duty ratio of 0.57. The proposed Q^4^HGC achieves a commendable *M/TCU* ratio of 1.25 while utilizing only 20 components. To standardize and eliminate the influence of duty ratio, the *M/TCU* ratio is calculated for all the converters at a fixed duty ratio of 50%. Surprisingly, except for the converters presented in^35,36^, the *M/TCU* ratio of all the other converters is either greater than or equal to 1. Both the converters in^35,36^ operate at the highest duty ratio value, which is responsible for their excellent *M/TCU* values. Expectedly, the proposed Q^4^HGC demonstrates the highest *M/TCU* value of 1.6 at the normalized duty ratio of 50%, followed by the converter outlined in^31^. Thus, the excellent voltage gain capability of the proposed Q^4^HGC and the judicious use of components in it are validated.

**Table 3:** Comparison of the proposed Q^4^HGC and some similar converters.

Attributes	Converter presented in						Proposed Q^4^HGC
	^31^	^34^	^35^	^36^	^37^	^39^	
*V*_*in*_	27 V	12 V	45 V	20	48	18 V	16 V
*V*_*0*_	400 V	170 V	800 V	650 V	650	380 V	400 V
*M*	14.81	14.16	17.78	32.5	13.5	21.1	25
*D*	0.5	0.48	0.632	0.63	0.48	*δ*_*0*_ = 0.50 *δ*_*3*_ = 0.57	*δ*_*1*_ = 0.50 *δ*_*3*_ = 0.46
*N*_*mag*_	2 (1 simple inductor and 2 CI)	3	3	3	3	4	5
*N*_*Sw*_	2	1	1	1	1	3	3
*N*_*Di*_	4	7	5	7	7	5	7
*TCU*	12	16	12	16	16	16	20
*M/TCU*	1.2	0.885	1.481	2.03	0.846	1.31	1.25
*M/TCU at δ = 0.5*	1.25	1	0.667	0.9375	1	1	1.6
Current stress of the switch (% of *I*_*in*_)	Min = 60% Max = 100%	Min = 194% Max = 194%	Min = 150% Max = 150%	Min = 100% Max = 100%	Min = 194% Max = 194%	Min = 39% Max = 100%	Min = 46% Max = 100%
Source current nature	Pulsating	Continuous with ripple	Pulsating	Pulsating	Pulsating	Ripple-free	Ripple-free
Gain extension technique	QBC with coupled inductor and DCM	Single Switch Cubic Boost Converter with SCs	Active inductor-capacitor-two diodes (LC2D) network	active switched inductor-capacitor network (SLCN)-based	active switched inductor-capacitor network (SLCN)-based	IBC with lift capacitor cascaded to QBC	Floating Capacitor based cubic Cell + IBC
Voltage gain function	Quadratic	Quadratic	Cubic	Cubic	Quartic	Cubic	Quartic
η (%)	94.9	–	91.6	90.04	95.48	95.6	92.7

*N*_*mag*_ no. of magnetic elements, *N*_*Sw*_ no. of switches, *N*_*Di*_ no. of diodes, *TCU* total components used.

### Voltage stress on the switches and diodes

**Fig. 18:**
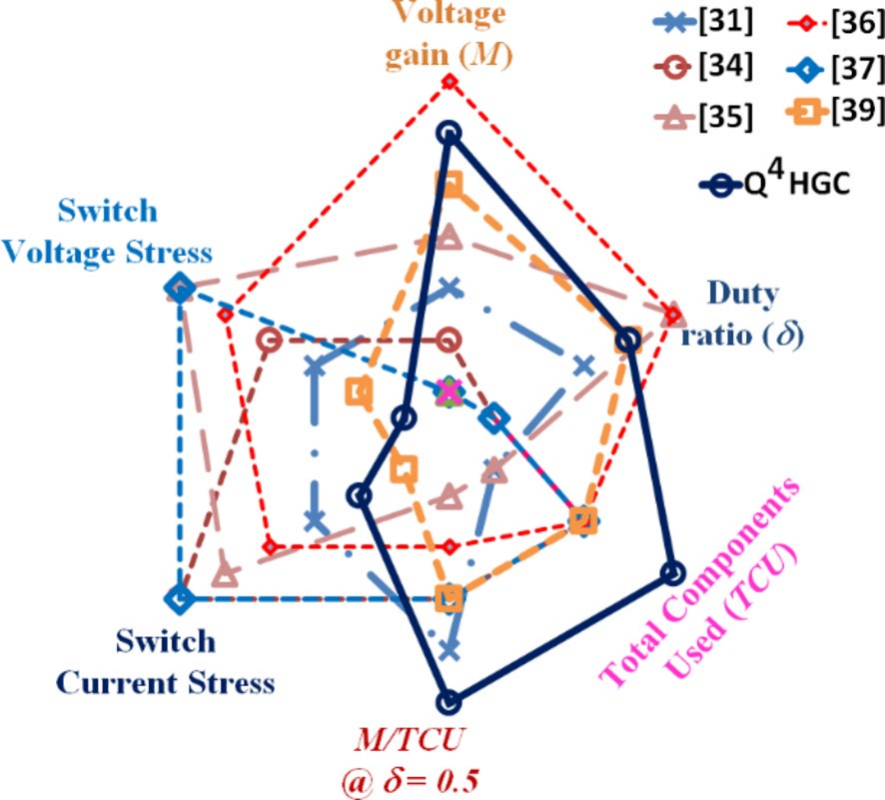
Radial chart depicting the attributes based on which the converters compared.

The proposed converter and the one detailed in^39^ share a common feature of employing three power switches, in contrast with the majority of compared converters, which utilize only one power switch. The converter described in^31^ utilizes two switches, notably with low voltage stress values. The voltage stress across the switch with a higher value is still only 38.75% of *V*_*0*_. Likewise, the converters presented in^34,36^ exhibit maximum switch voltage stress values that are well-below *V*_*0*_. Consequently, the converters employ MOSFETs with lower *R*_*DS−ON*_ values and achieve good efficiency values. The converters discussed in^35,37^ utilize only one power switch experiencing the same voltage stress of *V*_*0*_. In the proposed converter and the one described in^39^, two switches are subjected to the least voltage stress, representing only 8% and 9.4% of *V*_*0*_ respectively. In both these converters, one among the three switches experiences a voltage stress, which is the same as *V*_*0*_. Figure 18 portrays the comparative attributes as a radial chart.

**Fig. 19:**
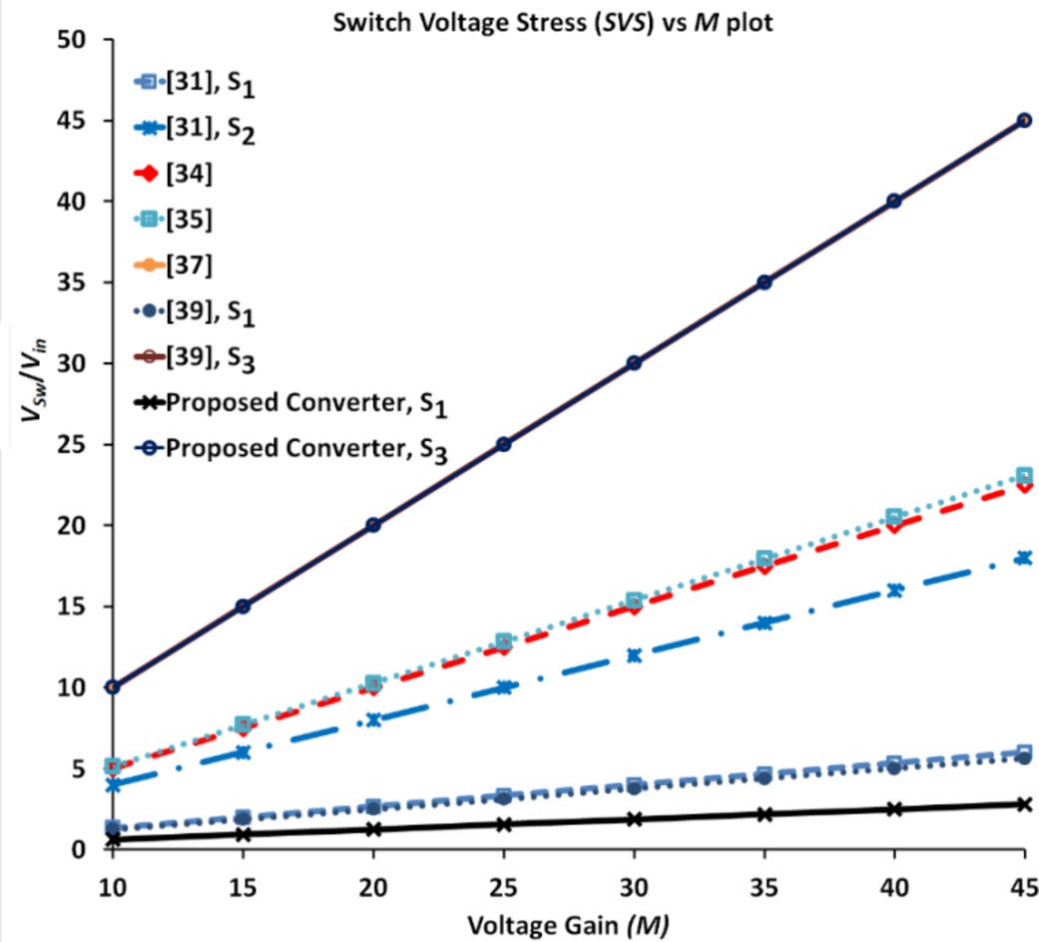
Variation of *SVS* versus *M* for all the converters compared in Table 4.

The normalized voltage stress value is obtained to showcase the advantageous features of the proposed converter. Several parameters are defined as follows. The switch voltage stress (*SVS*) is computed as *V*_*sw*_/*V*_*in*_ and presented as a comparative attribute. Figure 19 demonstrates the *SVS* variation of all the converters compared in Table IV. Two of the three switches employed in the proposed converter experience the least variations. The normalized diode voltage stress (*NDVS*) and normalised switch voltage stress (*NSVS*) refer to the ratio of maximum voltage across the diode to *V*_*0*_ and the switch to *V*_*0*_, respectively, total voltage stress (*TVS*) is defined as the sum of voltage stress across all the semiconductor devices. It is computed and expressed as a percentage of *V*_*0*_ as given by (48). To get an idea about the average voltage stress in the converter, the normalized TVS (*NTVS*) value is obtained and presented in (49).

48 $$TVS=\sum {\left( {\frac{{{V_{{D_i}}}}}{{{V_0}}}+\frac{{{V_{{S_i}}}}}{{{V_0}}}} \right)} \times 100$$

49 $$NTVS=\frac{1}{{{N_{devices}}}}\sum {\left( {\frac{{{V_{{D_i}}}}}{{{V_0}}}+\frac{{{V_{{S_i}}}}}{{{V_0}}}} \right)} \times 100$$

where ${V_{{D_i}}}$and ${V_{{S_i}}}$refers to the peak voltage stress of *i*th diode and switch respectively and *N*_*devices*_ refers to the number of switching elements.

Most of the converters have at least one diode and power switch that experiences a voltage stress that is equal to *V*_*0*_. Consequently, 4 out of 7 converters, including the proposed converter, exhibit a maximum *NDVS* and *NSVS* value of 100%. Converters presented in^34,37^ exhibit the least and second lowest *NDVS* values of 50% and 61%, respectively, whereas the converter presented in^31^ experiences the lowest *NSVS* of 38.75%. The converter presented in^31^ has the least *TVS* value since it exhibits the lowest *NSVS* and *NDVS* values.

**Fig. 20:**
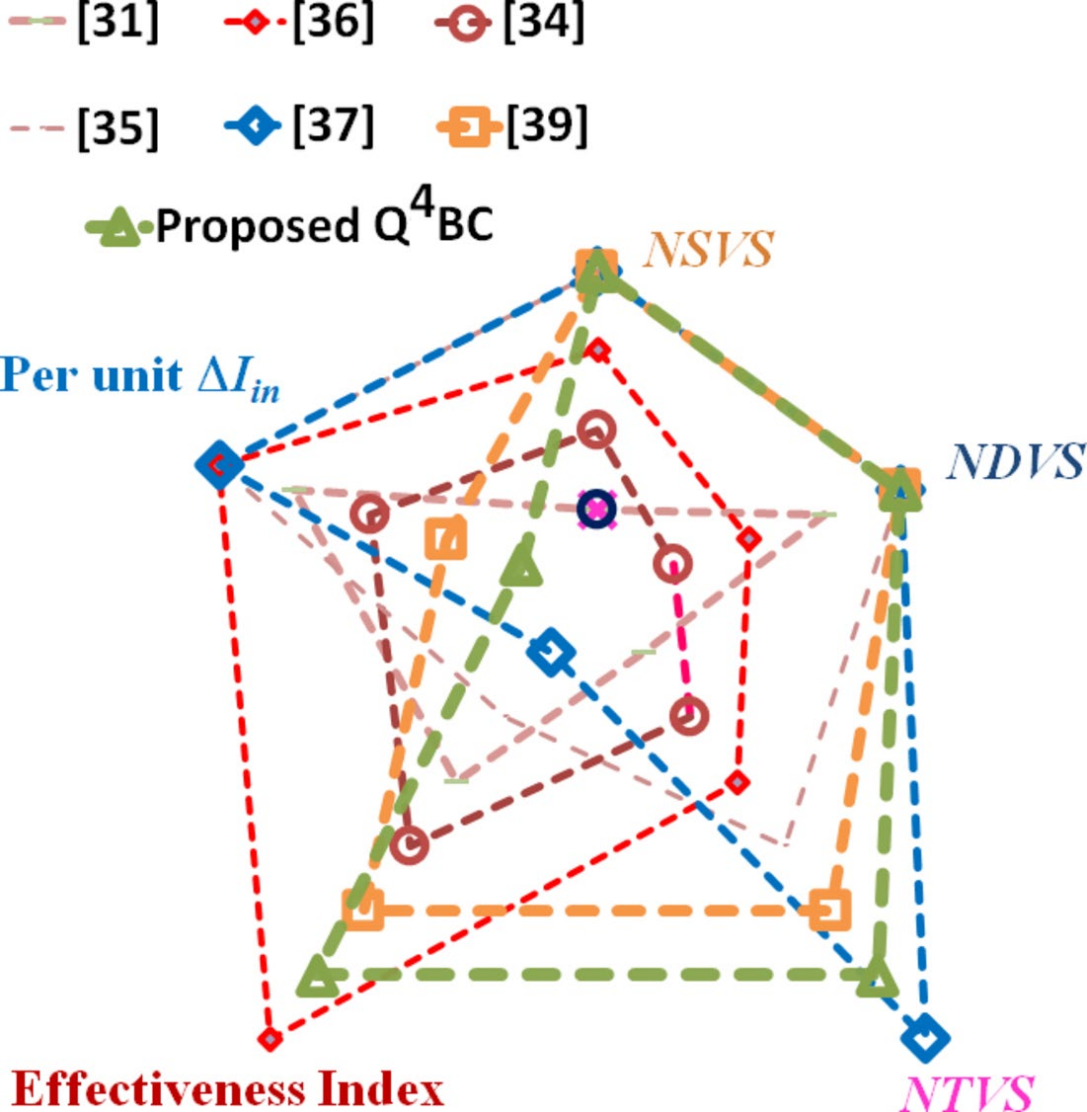
Radial chart depicting the voltage stress on the semiconductor devices used in the converters compared in Table 4.

To gain a clearer perspective on the average stress across components, the normalized total voltage stress (*NTVS*) is calculated and compared. The proposed converter stands out with the second lowest *NTVS* value of 40.4%, outperforming all other compared converters except the one in^31^, which has the lowest *NTVS* of 37.41%. However, it is important to note that although the switch of the converter presented in^31^ has reduced stress, it experiences significantly higher current stress. This could potentially lead to lower efficiency at higher power levels. To further evaluate the performance of these converters, a new term effectiveness index (*EI*) is introduced. The *EI* is defined as the ratio of *M* and *NTVS*. The proposed converter outperforms all the converters except the one presented in^36^, which has an *EI* of 0.72, compared to the proposed converter’s *EI* of 0.62. However, the converter in^37^ is operated at a duty ratio of 0.63. When the proposed converter’s duty ratio is matched, its *EI* value shoots up to 1.95 and outperforms the other converters by a huge margin. Thus, the judicious utility of all the components is justified. Figure 20 portrays the comparative metrics related to voltage stress on the semiconductor devices as a radial chart.

### Source current behaviour

Generally, the effective implementation of the MPPT algorithm often hinges on the current drawing profile of the intermediate high-gain converters.

**Table 4:** Comparative metrices related to voltage stress of the devices used in the proposed Q^4^HGC and some similar converters.

Attributes	Converter presented in						Proposed Q^4^HGC
	^31^	^34^	^35^	^36^	^37^	^39^	
SVS = *V*_*sw*_/V_in_	$$\begin{gathered} \frac{{{V_{{S_1}}}}}{{{V_{in}}}}=\frac{1}{{(1 - \delta )}}\, \hfill \\ \frac{{{V_{{S_2}}}}}{{{V_{in}}}}=\,\frac{{2 - D}}{{{{(1 - \delta )}^2}}}\, \hfill \\ \end{gathered}$$	$\,\frac{1}{{{{(1 - \delta )}^3}}}\,$	$\,\frac{1}{{{{(1 - \delta )}^3}}}\,$	$\,\frac{1}{{{{(1 - \delta )}^3}}}\,$	$\,\frac{1}{{{{(1 - \delta )}^4}}}\,$	$$\begin{gathered} \frac{{{V_{{S_1}}}}}{{{V_{in}}}}=\frac{{{V_{{S_2}}}}}{{{V_{in}}}}=\frac{1}{{(1 - \delta )}}\, \hfill \\ \frac{{{V_{{S_3}}}}}{{{V_{in}}}}=\,\frac{2}{{{{(1 - \delta )}^3}}}\, \hfill \\ \end{gathered}$$	$$\begin{gathered} \frac{{{V_{{S_1}}}}}{{{V_{in}}}}=\frac{{{V_{{S_2}}}}}{{{V_{in}}}}=\frac{1}{{(1 - \delta )}}\, \hfill \\ \,\frac{{{V_{{S_3}}}}}{{{V_{in}}}}=\frac{2}{{{{(1 - \delta )}^4}}}\, \hfill \\ \end{gathered}$$
*NSVS* (%)	38.75	50	100	61	100	100	100
*NDVS* (%)	92	50	100	61	100	100	100
*TVS* (%)	247.7	298	337	303	429	345	404
*NTVS* (%)	41.28	37.41	56.27	45.46	53.63	43.4	40.4
Effective ness index (*M/NTVS*)	0.36	0.38	0.32	0.72	0.25	0.49	0.62
Input current ripple (% of *I*_*in*_)	133	18	200	200	200	3.43	3.25

*SVS* switch voltage stress, *NSVS* normalized switch voltage stress, *NDVS* normalized diode voltage stress, *TVS* total voltage stress, *NTVS* normalized total voltage stress.

**Table 5:** Comparison of the proposed Q^4^HGC with some interleaved converters.

Attributes	Converter presented in						Proposed Q^4^HGC
	^8^	^9^	^10^	^29^	^38^	^39^	
*V*_*in*_	18 V	18 V	18 V	18 V	24 V	18 V	16 V
*V*_*0*_	380 V	380 V	380 V	380 V	380 V	380 V	400 V
*M*	21.11	21.11	21.11	21.11	15.833	21.1	25
*D*	0.5	0.5	0.5	0.5	0.65	*δ*_*0*_ = 0.50 *δ*_*3*_ = 0.57	*δ*_*1*_ = 0.50 *δ*_*3*_ = 0.46
*N*_*mag*_	2 CIs	2 CIs	2 CIs	2 CIs	2 CIs	4	5
*N*_*Sw*_	2	2	2	2	2	3	3
*TCU*	16	18	14	18	14	16	20
*M/TCU*	1.32	1.17	1.57	1.17	1.13	1.31	1.25

**Fig. 21:**
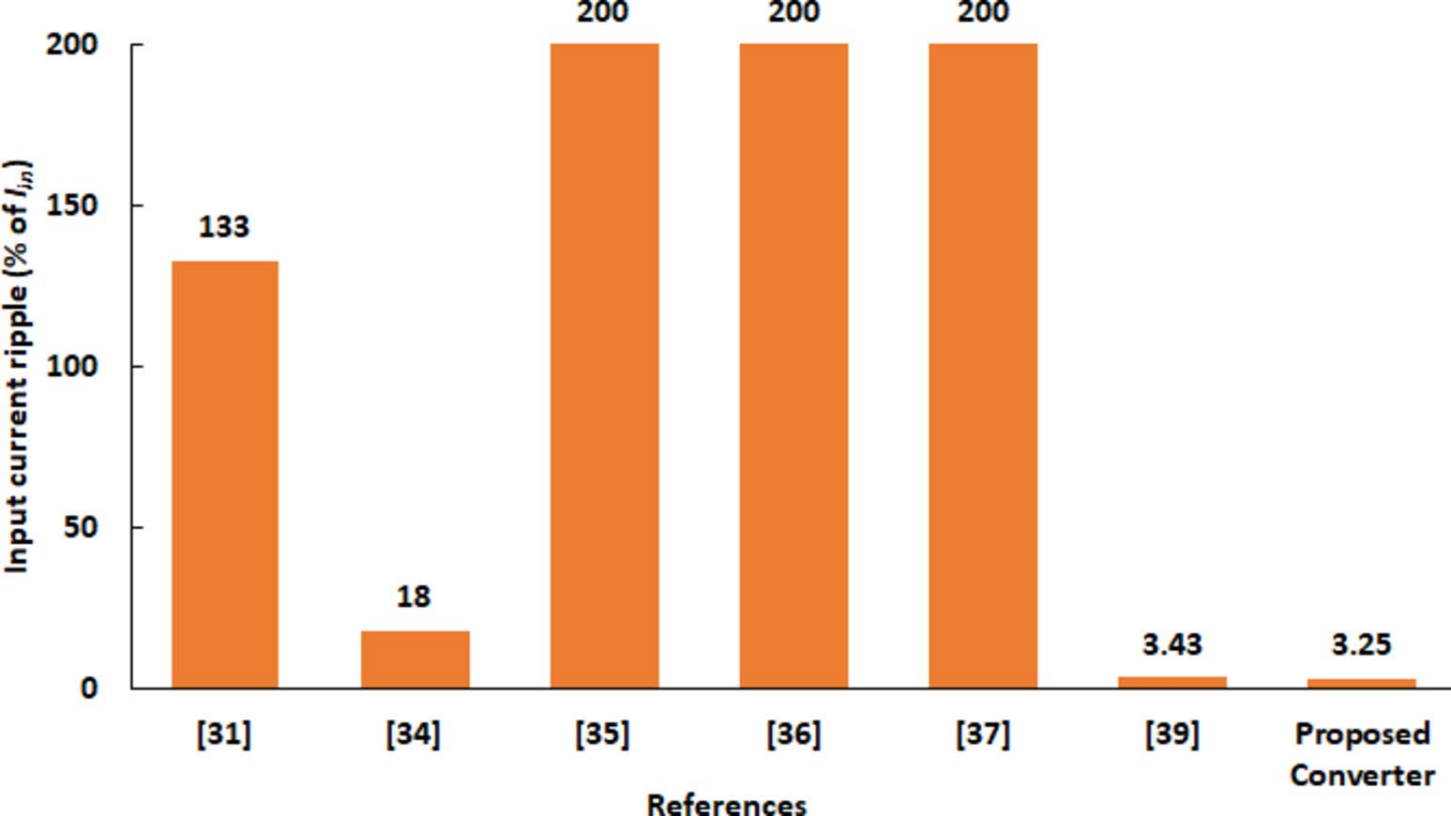
Input current ripple values (in percentage) of the converters compared in Table 4 and portrayed as a bar graph.

In PV applications, a converter that draws smooth and ripple-free current from the input port is preferred. Among the converters compared in Table 4, only the proposed Q^4^HGC and the one described in^39^ draw smooth and ripple-free current from the input. Additionally, the converter detailed in^34^ demonstrates continuous input current with controllable ripple, dictated by the inductance. All the other converters draw pulsating current from their respective input ports. Figure 20 pictorially depicts the key attributes of all the converters compared in Table 4. The input current ripple profiles of all the converters are portrayed in Fig. 21. Table 5 presents the comparative attributes of some interleaved high-gain DC-DC converters and the proposed Q4HGC. The beneficial features, viz., high voltage gain capability and the component utility factor of the proposed are observed.

## Conclusion

In this paper, a non-isolated high-gain DC-DC converter with quartic voltage gain capability was introduced and discussed. The proposed converter was synthesized from a two-phase IBC structure with a voltage lift capacitor, and its voltage gain was significantly enhanced by adopting a floating capacitor-based cubic voltage gain cell. The experimental results obtained from a laboratory prototype version of the proposed converter confirmed its excellent voltage gain capability. Under practical conditions, the converter yielded a voltage gain of 25 (16 V input to 400 V output) and delivered 150 W to the load at an impressive efficiency of 92.7%. The proposed Q^4^HGC employed three switches, and the voltage stress on two switches was just 8% of the output voltage due to the adopted gain extension concept. The voltage stress on five out of the seven diodes was less than 50% of the output voltage; only the output diode was subjected to a higher voltage stress level. Since an interleaving mechanism was adopted, the converter drew smooth and ripple-free current from the input port; the current stress on the switches was also significantly reduced. By implementing a closed-loop control, the output voltage of the Q^4^HGC was regulated. When the input voltage and load current underwent step changes over a wide range, the proposed converter responded swiftly, and its output voltage was restored to 400 V quickly with minimal undershoots and overshoots. In fact, the maximum voltage gain of the proposed converter momentarily increased to 37 while the switches were still operated at safe duty ratio values. To appreciate the beneficial features of the proposed converter, it was compared with some state-of-the-art converters possessing quadratic, cubic, and quartic voltage gain capabilities. The proposed converter excels in terms of higher voltage gain capability, better utilization of components, reduced voltage stress on the devices, and ripple-free input current operation. The converter possesses a common-ground connection between the input and output ports; it is an additional advantage for PV applications. Due to its salient advantageous features, the proposed Q^4^HGC is likely to be a good candidate topology for interfacing the low-voltage PV input with the high-voltage DC bus. Further, by incorporating open-circuit, short-circuit, and other protection mechanisms, the converter could be employed in a DC microgrid.

## Figures and Tables

**Fig. 22:**
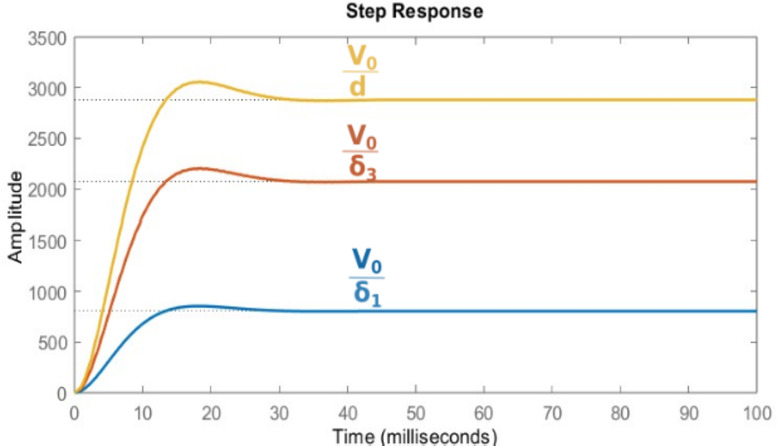
Step responses of the converter obtained from output voltage versus individual duty ratio of the switches and the overall duty ratio.

**Fig. 23:**
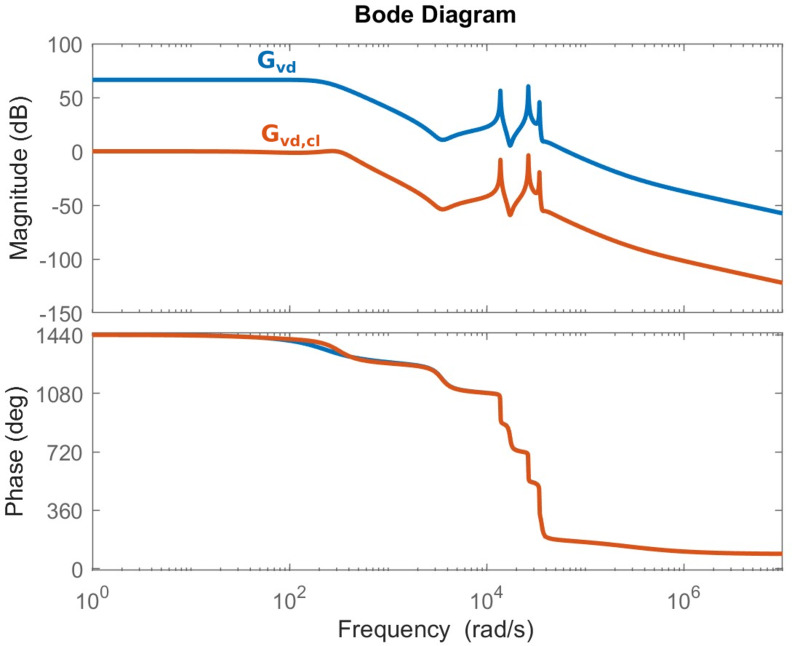
Step responses of the converter obtained from output voltage versus individual duty ratio of the switches and the overall duty ratio.

## Data Availability

All data generated or analysed during this study are included in this published article.

## Appendix

### State-space analysis and small signal model of the proposed converter

In this section, the focus lies in representing the low-frequency characteristics and response to small-signal variations using a state-space model. The well-known input and output relations in state-space form are given by (50), (51).

50 $$\dot {x}=Ax+Bu$$

51 $$y=Cx+Du$$

The voltage across the capacitors and current through inductors are chosen as state variables. The ON-state resistance of the diodes and magnetic elements is ignored while the loop resistances (r_1_ and r_2_) are included. The state variables are represented by the equation (52).

52 $$x={\left[ {{I_{{L_1}}}\,\,\,\,{I_{{L_2}}}\,\,\,{I_{{L_3}}}\,\,\,{I_{{L_4}}}\,\,\,{I_{{L_5}}}\,\,\,{V_{{C_{Lift}}}}\,\,{V_{{C_1}}}\,\,\,{V_{{C_2}}}\,\,\,{V_{{C_3}}}\,\,\,{V_{{C_0}}}} \right]^T},\;{\text{y}}\,=\,{v_0},{\text{ and}}\;u\,=\,{v_{in}}$$

Using the equations obtained during the operating modes and expressing them in state-space form, the state model of the system is derived and presented in (53)–(56). The two transfer functions viz., output voltage to duty ratio (*G*_*vd*_) and output voltage to input voltage (*G*_*vg*_) are determined and expressed using (55) and (56). Since all the poles of the transfer function lie of the left half of the s-plane, the proposed converter is stable. The control to output transfer functions of the system are derived by fixing one duty ratio a constant and varying the other.

53 $${\mathbf{A}}=\left( {\begin{array}{*{20}{c}} { - \frac{{r1}}{{{L_1}}}}&0&0&0&0&{ - \frac{{1 - {\delta _1}}}{{{L_1}}}}&0&0&0&0 \\ 0&{ - \frac{{r1}}{{{L_2}}}}&0&0&0&{\frac{{1 - {\delta _1}}}{{{L_2}}}}&{ - \frac{{1 - {\delta _1}}}{{{L_2}}}}&0&0&0 \\ 0&0&{ - \frac{{r2}}{{{L_3}}}}&0&0&0&{\frac{{{\delta _3}}}{{{L_3}}}}&{ - \frac{{1 - {\delta _3}}}{{{L_3}}}}&0&0 \\ 0&0&0&{ - \frac{{r2}}{{{L_4}}}}&0&0&{\frac{{{\delta _3}}}{{{L_4}}}}&{\frac{{{\delta _3}}}{{{L_4}}}}&{ - \frac{{1 - {\delta _3}}}{{{L_4}}}}&0 \\ 0&0&0&0&{ - \frac{{r2}}{{{L_5}}}}&0&{\frac{1}{{{L_5}}}}&{\frac{1}{{{L_5}}}}&{\frac{1}{{{L_5}}}}&{ - \frac{{1 - {\delta _3}}}{{{L_5}}}} \\ {\frac{{1 - {\delta _1}}}{{{C_{{\text{lift}}}}}}}&{ - \frac{{{\delta _1}}}{{{C_{{\text{lift}}}}}}}&0&0&0&0&0&0&0&0 \\ 0&{\frac{{{\delta _1}}}{{{C_1}}}}&{ - \frac{{{\delta _1}}}{{{C_1}}}}&{ - \frac{{{\delta _1}}}{{{C_1}}}}&{ - \frac{1}{{{C_2}}}}&0&0&0&0&0 \\ 0&0&{\frac{{1 - {\delta _3}}}{{{C_2}}}}&{ - \frac{{{\delta _3}}}{{{C_2}}}}&{ - \frac{1}{{{C_2}}}}&0&0&0&0&0 \\ 0&0&0&{\frac{{1 - {\delta _3}}}{{{C_3}}}}&{ - \frac{1}{{{C_3}}}}&0&0&0&0&0 \\ 0&0&0&0&{\frac{{1 - {\delta _3}}}{{{C_0}}}}&0&0&0&0&{ - \frac{1}{{{R_0} \cdot {C_0}}}} \end{array}} \right)$$

54 $${B_\delta }={\left[ {\begin{array}{*{20}{c}} {\frac{{{V_{{C_{Lift}}}}}}{{{L_1}}}}&{ - \frac{{{V_{{C_{Lift}}}}+{V_{{C_1}}}}}{{{L_2}}}}&{\frac{{{V_{{C_1}}}+{V_{{C_2}}}}}{{{L_3}}}}&{\frac{{{V_{{C_1}}}+{V_{{C_2}}}+{V_{{C_3}}}}}{{{L_4}}}}&{\frac{{{V_{{C_o}}}}}{{{L_5}}}}&{ - \frac{{{I_{{L_1}}}+{I_{{L_2}}}}}{{{C_{Lift}}}}}&{\frac{{{I_{{L_2}}} - {I_{{L_3}}} - {I_{{L_4}}}}}{{{C_1}}}}&{ - \frac{{{I_{{L_3}}} - {I_{{L_4}}}}}{{{C_2}}}}&{ - \frac{{{I_{{L_4}}}}}{{{C_3}}}}&{ - \frac{{{I_{{L_5}}}}}{{{C_o}}}} \end{array}} \right]^T}$$

55 $$B={\left[ {\begin{array}{*{20}{c}} {\frac{1}{{{L_1}}}}&{\frac{1}{{{L_2}}}}&0&0&0&0&0&0&0&0 \end{array}} \right]^T}$$

56 $$C={\left[ {\begin{array}{*{20}{c}} 0&0&0&0&0&0&0&0&0&1 \end{array}} \right]^T} \, \text{and}\,D \text{is a null-matrix}$$

57 $$\,\frac{{{v_{0\,}}}}{{{{\hat {v}}_{in}}}}\,=\frac{{4.69 \times {{10}^{14}}{s^6}+3.06 \times {{10}^{17}}{s^5}+1.59 \times {{10}^{24}}{s^4}+5.94 \times {{10}^{26}}{s^3}+1.54 \times {{10}^{33}}{s^2}+1.83 \times {{10}^{35}}s+3.19 \times {{10}^{41}}}}{{{s^{10}}+999.90{s^9}+3.59 \times {{10}^9}{s^8}+2.83 \times {{10}^{12}}{s^7}+4.32 \times {{10}^{18}}{s^6}+2.58 \times {{10}^{21}}{s^5}+1.94 \times {{10}^{27}}{s^4}+8.42 \times {{10}^{29}}{s^3}+2.36 \times {{10}^{35}}{s^2}+7.30 \times {{10}^{37}}s+1.27 \times {{10}^{40}}}}{\text{ }}$$

The closed-loop control to output transfer function of the proposed Q^4^-HGC is achieved by fixing *δ*_*1*_ constant while varying *δ*_*3*_ and vice versa. The two transfer functions are given by (56) and (57).

58 $$\frac{{{v_0}}}{{{{\overset{\lower0.5em\hbox{$\smash{\scriptscriptstyle\frown}$}}{\delta } }_3}}}=\frac{{ - 1.316 \times {{10}^4}{s^9}+3.737 \times {{10}^9}{s^8} - 5.831 \times {{10}^{13}}{s^7}+1.172 \times {{10}^{19}}{s^6} - 7.929 \times {{10}^{22}}{s^5}+1.07 \times {{10}^{28}}{s^4} - 3.357 \times {{10}^{31}}{s^3}+2.328 \times {{10}^{36}}{s^2} - 2.94 \times {{10}^{39}}s+2.632 \times {{10}^{43}}}}{{{s^{10}}+999.9{s^9}+3.587 \times {{10}^9}{s^8}+2.825 \times {{10}^{12}}{s^7}+4.317 \times {{10}^{18}}{s^6}+2.579 \times {{10}^{21}}{s^5}+1.944 \times {{10}^{27}}{s^4}+8.417 \times {{10}^{29}}{s^3}+2.363 \times {{10}^{35}}{s^2}+7.297 \times {{10}^{37}}s+1.268 \times {{10}^{40}}}}$$

59 $$\frac{{{v_0}}}{{{{\overset{\lower0.5em\hbox{$\smash{\scriptscriptstyle\frown}$}}{\delta } }_1}}}=\frac{{1.116 \times {{10}^{11}}{s^7}+1.511 \times {{10}^{16}}{s^6}+3.878 \times {{10}^{20}}{s^5}+5.108 \times {{10}^{25}}{s^4}+3.857 \times {{10}^{29}}{s^3}+4.945 \times {{10}^{34}}{s^2}+8.173 \times {{10}^{37}}s+1.022 \times {{10}^{43}}}}{{{s^{10}}+999.9{s^9}+3.587 \times {{10}^9}{s^8}+2.825 \times {{10}^{12}}{s^7}+4.317 \times {{10}^{18}}{s^6}+2.579 \times {{10}^{21}}{s^5}+1.944 \times {{10}^{27}}{s^4}+8.417 \times {{10}^{29}}{s^3}+2.363 \times {{10}^{35}}{s^2}+7.297 \times {{10}^{37}}s+1.268 \times {{10}^{40}}}}{\text{ }}$$

From the transfer functions, the step responses of the proposed converter are obtained and plotted in Fig. 22. The converter exhibits a quick response and settles within 30 ms.

The control of the proposed converter is achieved by varying δ3 and the corresponding transfer function is given by (57). The open-loop response of the system was inherently unstable, with a gain margin of 0.56 dB and a phase margin of − 6.97°, indicating poor stability and a tendency toward instability. To improve the performance an PI controller is designed is MATLAB PID tuner. The designed PID controller has proportional and integral gains given by Kp = 0.000593 and Ki = 0.0697. After implementing the PID controller, significant improvements were observed in the closed-loop system. The gain margin increased to 25.17 dB, and the phase margin improved to 119.98°, indicating a much more stable and robust system response. The integral action in the PID controller effectively mitigates steady-state errors, while the proportional action enhances the dynamic response, ensuring improved performance and stability under varying system conditions. The open-loop and closed-loop Bode diagrams are presented in Fig. 23.
